# The exclusive effects of chaperonin on the behavior of proteins with 5_2_ knot

**DOI:** 10.1371/journal.pcbi.1005970

**Published:** 2018-03-16

**Authors:** Yani Zhao, Pawel Dabrowski-Tumanski, Szymon Niewieczerzal, Joanna I. Sulkowska

**Affiliations:** 1 Centre of New Technologies, University of Warsaw, Warsaw, Poland; 2 Institute of Physics, Polish Academy of Sciences, Warsaw, Poland; 3 Faculty of Chemistry, University of Warsaw, Warsaw, Poland; Koç University, TURKEY

## Abstract

The folding of proteins with a complex knot is still an unresolved question. Based on representative members of Ubiquitin C-terminal Hydrolases (UCHs) that contain the 5_2_ knot in the native state, we explain how UCHs are able to unfold and refold *in vitro* reversibly within the structure-based model. In particular, we identify two, topologically different folding/unfolding pathways and corroborate our results with experiment, recreating the chevron plot. We show that confinement effect of chaperonin or weak crowding greatly facilitates folding, simultaneously slowing down the unfolding process of UCHs, compared with bulk conditions. Finally, we analyze the existence of knots in the denaturated state of UCHs. The results of the work show that the crowded environment of the cell should have a positive effect on the kinetics of complex knotted proteins, especially when proteins with deeper knots are found in this family.

## Author Summary

Self-tying of knotted proteins remains a challenge both for theoreticians and experimentalist. In this work, we study the proteins with complex, the 5_2_ knot, in a bulk and confined within a chaperonin box. We show that in our model we recreate the experimental results, identify two topologically distinct folding pathways and explain the beneficial role of confinement for complex knotted proteins. Encapsulation provides a possibility to fold via alternative pathway—folding via trefoil intermediate knot (N-terminal pathway) from entropic reason while folding via the C-terminal (direct tying) appears with the same probability. The results of this work show, how crowded environment in the real cell may enhance self-tying of proteins. The results are also the first step to the identification of possible oligomerization-prone forms of UCHs, which may cause neurodegenerative diseases.

## Introduction

The role of knots in protein structures is still not fully understood. The topological complexity induces stability to the structure [1, 2] and enforces local motifs favorable for active sites of enzymes [3]. The latter fact may explain, why over 80% of known knotted proteins are enzymes with the active site located at the entangled region [4]. Nevertheless, folding process of knotted proteins is a fundamental and still not solved problem.

**Fig 1:**
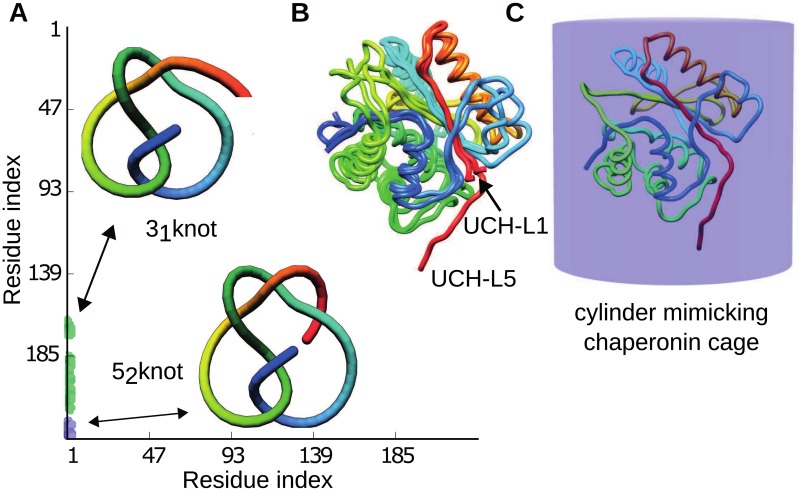
Molecular structures and a topological fingerprint for Ubiquitin C-terminal Hydrolases, UCH. A: The topological fingerprint—presented as a matrix—for UCH shows that this protein forms the Gordian knot (5_2_) as a whole but some of its subchains form two trefoil slipknots (described in detail in the text). B: Ribbon representation of backbone superposition of UCH-L1 (PDB code 2LEN and 3IRT) and UCH-L5 (PDB code 4I6N), where red and blue color indicate the N- and C-terminus, respectively. C: Model of the chaperonin cage, a cylindrical box (closed from both sides, with height equal to the diameter), which encapsulates the UCH-L5 protein.

One of the families of knotted proteins is Ubiquitin C-terminal Hydrolase (UCH) of which the characteristic feature is the presence of a complex topological fingerprint 5_2_3_1_3_1_ [4] as shown in the Fig 1. This means that the entire protein forms a 5_2_ knot as a whole, but some of its subchains form two trefoil knots (see Table 1). Each entry in the matrix indicates the knot type, formed by one continuous subchain, by one particular color; e.g. the unknot is denoted in white. Each such subchain starts with the N-terminal amino acid at position *x* and ends with the C-terminal amino acid at position *y*, and the corresponding colored entry in the matrix is shown in position (*x*, *y*) (along respectively horizontal and vertical axes). Specifically, one can trace what is the topology of the subchain with one end in N-terminus. Pictorially, this is represented as the traveling down of the left-most vertical line in the matrix in Fig 1). In the beginning, successive subchains are unknotted, however reaching at least Ile163 the subchain becomes trefoil knotted (first green patch in the matrix). The subchain Met1-Tyr173 is still knotted, however then the chain winds back forming a slipknot loop and when the end of the subchain is in-between Glu174 and Pro180 (parts of C-terminal *β*-strands), such subchain is unknotted (the break between the green patches). Next, the subchain starting in N-terminus and ending in-between residues Pro182 and Ala216 is again trefoil knotted (bottom green patch) and finally the whole chain is 5_2_ knotted (blue patch). The 5_2_3_1_3_1_ fingerprint is unique and conserved in all UCH members, which are separated by billion years of evolution and exhibit a very low sequence similarity (below 30%) [5]. Notably, the formation of the larger trefoil results in the formation of the inner-most (dipper) trefoil knot. Therefore, in subsequent analysis by “formation of 3_1_ knot”, we mean the formation of the larger (and hence both) trefoil knot.

**Table 1:** Protein structures investigated in this work. *L*_*seq*_ denotes the number of residues in the structure, “cont.” the number of native contacts, *N*_tail_ and *C*_tail_ the length of N- and C-terminal 5_2_ knot tails. The knotted core of the innermost 3_1_ knot inside 5_2_ knot is also shown. PDB code 4WLR denotes protein with the deepest 5_2_ knot in the UCH family. More detailed comparison incl the sequence similarity is given in SI.

PDB id	UCH type	organism	*L*_*seq*_	cont.	knot location		*N*_tail_	*C*_tail_
					3_1_	5_2_		
3IRT	L1	Homo sapiens	223	454	6-162	5-220	4	2
2LEN	L1	Homo sapiens	231	387	5-163	6-220	5	10
4I6N	L5	Trichinella spiralis	229	470	12-166	12-225	11	3
4I6N-m	–	–	222	457	5-159	5-218	4	3
4WLR	L5	Mus musculus	313	860	6-160	5-219	4	94

The UCH superfamily is a group of deubiquitinating enzymes (DUBs). Their exact substrates have not yet been determined, however it seems that the role of UCHs is to detach the ubiquitin from small nucleophiles. Four of the UCH family members exist in humans: UCH-L1, UCH-L3, human UCH-L5 (UCH37) and BAP-1. They share a high degree of homology in their catalytic domains [6], surrounded by the deepest 3_1_ knot. Moreover, UCHs have a tissue-specific expression in complex organisms such as humans and their activity is crucial from the therapeutical point of view. For example, UCH-L3 has been shown to be upregulated in breast cancer tissues [7], and a high expression of UCH-L5 is significantly associated with poor prognosis in human epithelial ovarian cancer [8]. On the other hand, UCH-L1 is one of the most common proteins in human brain (composing up to 1-2% of the brain the total protein content [9]), and it is highly expressed in pancreatic [10], esophageal [11], prostate [12], medullary thyroid [13], colorectal carcinomas [14] and HPV16-transformed cells [15]. Its misfolded forms were connected with neuronal disorders such as Parkinson’s, Huntington’s and Alzheimer’s diseases [16], which justifies the importance of studying the UCH folding process.

In general, it is expected that folding of knotted proteins is governed mainly by the depth of the knot and the complexity of the topological fingerprint [17, 18]. Self-tying was observed theoretically for the smallest knotted proteins with DNA binding motif and a rather shallow 3_1_ knot [19, 20]. They mainly fold by a slipknot conformation [17, 19]. Similarly, it was shown that proteins with a deep trefoil knot, such as YibK and YbeA, can self-tie [21]. The theoretical results obtained for these proteins with a structure based model additionally revealed that a knotting event is a rate-limiting step [22] and the folding efficiency can be controlled by non-native contacts [23] or consideration of cotranslational on-ribosome folding [24]. For the protein with a 6_1_ knot, DehI [25], there were only a few successful folding pathways observed theoretically. Surprisingly, this protein folds via a simple mechanism: a large twisted loop formed on the backbone flips over another protein fragment previously arranged in a twisted loop, and in consequence, the six-fold knot is created in a single movement. Even though this protein is prone to aggregate, the experimental data support this mechanism [26]. These results suggest that bulk structure-based models can be used to investigate knotted proteins.

On the other hand, experimental data show that knotting process of trefoil-knotted YibK and YbeA bacterial proteins can be specifically and significantly accelerated by the GroEL-GroES chaperonin complex [21] encapsulating the folding protein. This agrees with the theoretical investigation showing that knotting probability of polymers increases in confinement [27]. Only due to the encapsulation (following [28]), successful reversible folding was observed for members of knotted proteins with DNA binding motif VirC and DndE [29]. It is then natural to expect, that chaperonins encapsulating proteins may also facilitate folding and self-knotting of eucaryotic UCHs, although no experimental result in this topic is available yet. Nevertheless, one has to bear in mind, that encapsulation is the simplest possible model of chaperonin, lacking many “biological features”—specific binding to the cage, chaperonin conformational changes, etc.

However, any theoretical results have to be confronted with the experimental data concerning UCHs folding. It has been shown already that UCHs can fold and refold reversibly in two parallel pathways, each consisting of one slow and one fast phase, as determined from chevron plot [30, 31]. Despite a common mechanism, folding processes of different UCHs is characterized by different kinetic parameters. Such differences can stem from a various depth of knots in UCH family [4], ranging from rather shallow (from the N-terminus) to the deep knot, which we just found, as shown in Table 1. The most attention-drawing are the S18Y and I93M mutations, which were found to modify (either decrease or increase) the risk of Parkinson’s disease [32], and the intermediates on folding pathway, as these are especially prone to oligomerization [33]. However, the results concerning these mutations are variable and differ in different studies. Despite the successful assignment of the majority of signals in NMR spectrum of the UCH-L1 [34], and in spite of studies of its tryptophan variants [33], the exact conformation of intermediates is still unclear. This may be due to a broad structural plasticity around the intermediate states [30]. The self-tying was postulated via the direct knotting event in accordance with the theoretical study of the on-lattice model of a designed by hand heteropolymer chain with 5_2_ knot [35]. However, the optical tweezers stretching experiment showed that the threading significantly decelerates the folding [36]. Still, because the topology cannot be detected in the *in vitro* experiment, the mechanism of knot tying remains unresolved.

In this study, we asked following questions: What is the difference between the two experimentally observed, parallel folding pathways? What is the influence of a chaperonin cage (confinement) on the folding and self-tying of UCHs? And more generally, what are the dynamical properties of UCH in a bulk and in a confinement? To answer these questions, we performed a comprehensive study of representative UCH members in a model of chaperonin cage (mimicked by repulsive cavity) and in the bulk, using structure-based model simulations. To ensure robustness of the results, we investigated proteins from different organisms, with low sequence similarity, and different depth of the knot. The results show that the structure-based model was sufficient to knot and unknot each of the studied proteins with and without the presence of confinement. However, only in the confinement, the simulations in transition temperature were accessible. We performed a comprehensive analysis of knot occurrence during simulations, resulting in the identification of two topologically distinct pathways. To relate our results to the experiment, we reproduce the chevron plot for a representative protein member of UCH family, revealing the existence of fast and slow phases. Next, we studied short-lived knots on the folding and the unfolding pathway and revealed for the first time the existence of random knots in the unfolded protein chain. To our knowledge, this is the first theoretical study with a direct investigation of the influence of the excluded volume on proteins containing complex knots.

## Materials and methods

### Protein structures

To obtain robust results, three sequentially different members of UCH family denoted with their PDB codes 3IRT (UCH-L1), 2LEN (UCH-L1), and 4I6N (UCH-L5) were studied. Additionally, to check an influence of the length of knot tails, we constructed *in silico* mutant of 4I6N, denoted as 4I6N-m, obtained by removing 7 residues from the N-terminus. All investigated structures feature a left-handed 5_2_ knot and complex topological fingerprint 5_2_3_1_3_1_. Alignment of the 3-dimensional structures of the studies proteins is shown in the Fig 1 and their most important topological and structural information is summarized in Table 1. Further structural and sequential comparison of chosen structures is presented in S1 Appendix, part 1. Not determined region (amino acids 142–152) in the structure of 4I6N was repaired using Modeller software [37], where the model with the lowest DOPE potential (Discrete Optimized Protein Energy) was chosen. The DOPE potential is one of the quantities assessing the structure correctness [38].

### The model and simulations

The dynamics of investigated structures was studied in structure based C*α* model [39, 40] with standard parameters as proposed by the SMOG server [41]. The model included bonded interactions (bonds, planar and dihedral angles), bead excluded volume (Lennard-Jones repelling part) and non-bonded interactions described with a 10-12 Lennard-Jones potential. The non-bonded attraction was applied between residues forming contacts in the native structure, as defined in [42]. The number of the native contacts for all considered proteins is presented in Table 1. The folding/unfolding transitions were studied through constant temperature molecular dynamics simulations with the Nose-Hoover thermostat (coupling constant eq. 0.025) using Gromacs v4.5.4 package [43]. Temperature $\tilde{T}$ in Gromacs is defined by the equation $\tilde{T}=\left(k_{b}T\right)/(\epsilon {\tilde{k}}_{b}$), where ${\tilde{k}}_{b}=0.00831451$. Through the text, the temperature is denoted simply as T and the Boltzman constant ${\tilde{k}}_{b}$ as *k*_*b*_. There were performed 200 simulations for each structure and temperature. The number of steps was in the range 10^7^ − 1.6⋅10^9^ steps depending on the condition.

### Simulation of the confinement

The confinement is represented by a cylinder (Fig 1) with a diameter equal to its height equal 6.0 nm [44], introduced into the system as in [29]. The interactions between the inner wall of the cylinder and protein are purely repulsive (only a confinement effect). Such model was previously used to study confinement or crowding effect on protein folding [44–46].

### Topological analysis

The data concerning the position of the knotted core and the length of the knot tails (Table 1) are taken from KnotProt server [4]. The knot type of each of the subchains of the protein is determined using the implementation of the HOMFLY-PT polynomial [47–49] and the chain closing method as in [5, 50]. The same algorithm was used to detect the entanglement along the protein backbone during simulations. The knot was regarded as present in the simulation if it was detected for at least 5 consecutive frames.

### Reaction coordinates, (un)folding pathways

The similarity to the native state was measured by the fraction of native contacts, *Q*. At given conformation, each native contact was regarded as present, if the distance between a pair of *C*_*α*_ atoms was less than 1.2 times their native distance. The untied structure was regarded as unfolded, if *Q* < 0.2. By unfolding pathway, we mean the shortest part of trajectory connecting knotted structure with *Q* > 0.9 and unfolded structure. By folding pathway, we mean the shortest part of the trajectory connecting unknotted structure with *Q* < 0.4 and knotted structure with *Q* > 0.9. Folding trajectories start from one of the previously generated 100 unknotted conformations with *Q* < 0.2. All initial structures belong to separate clusters with 0.1 nm cutoff, to remove any possible bias.

### Visualisation

The structures were visualized using UCSF Chimera [51].

## Results

The landscape of successful folding pathways that leads to the correctly knotted native conformation is observed for each of the proteins, in both conditions through our simulations. As the folding of all UCHs is similar [31], we concentrated our study on the most commonly analyzed protein—UCH-L1 (PDB code 3IRT), comparing results with other UCHs when needed. Our results naturally split into three parts: description of folding/unfolding landscape, kinetics of the process and an analysis of the random and short-lived knots.

### The landscape of folding and unfolding of 5_2_ knotted proteins from the UCH family

#### Folding of UCHs

**Fig 2:**
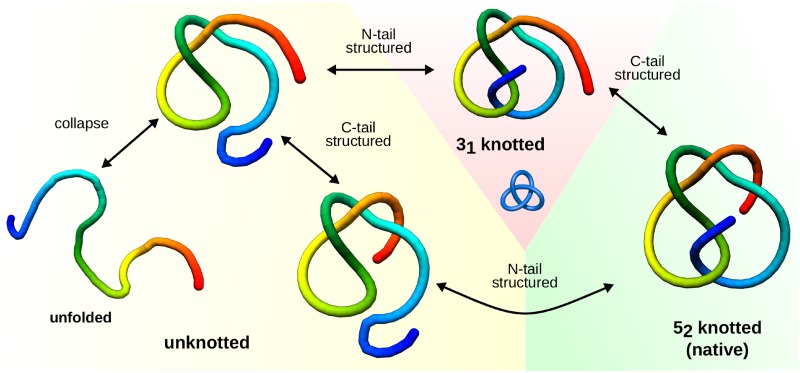
Schematic representation of possible folding pathways of UCHs. The folding starts with a collapse, next, depending on which terminus becomes structured first, two topologically distinct pathways are possible. Lower arrows: the pathway where N-terminus is structured after C-terminus (*F*_*N*_), protein self-ties in one step (direct 0_1_ → 5_2_ transition). The upper arrows: pathway where C-terminus is structured last (*F*_*C*_)—the protein follows 0_1_ → 3_1_ → 5_2_ pathway, where in the 3_1_ knotted intermediate the N-terminus is in its native position, while C-terminus is not. Different background denotes different topology.

Folding of UCHs is known to follow two parallel pathways [30, 31]. In our simulations, data analysis of time evolution of the topology revealed also two, topologically distinct pathways, shown schematically in Fig 2. The pathways in general differ in the order of the protein tails’ secondary and tertiary structure formation:

- *F*_*N*_—the N-terminus is structured last, in particular after C-terminus (resulting in a direct 0_1_ → 5_2_ transition);
- *F*_*C*_—the C-terminus is structured last, in particular after N-terminus—the protein follows 0_1_ → 3_1_ → 5_2_ pathway, where in the 3_1_ knotted intermediate the N-terminus is in its native position, while C-terminus is not.

The existence of 3_1_ knotted intermediate was questioned before as in principle it should require two threading events (of both termini), being hence entropically unfavorable [33]. Observe however, that actually two threadings are required independently on the pathway, however, they can be realized in a relatively easy way, if the protein is not yet collapsed. In particular, the tail may first form some of its secondary structure, and then the knotting loop may build upon the structured terminus (“squeezing” the loop around it). The existence of the self-tying mechanism in the bulk, realized by two parallel pathways, is clearly in agreement with the experimental results.

To extract the dominating folding mechanism, we determined the probabilities (frequency of occurrences) of each pathway (*F*_*N*_ and *F*_*C*_). The results for various temperatures in both conditions are presented in Table 2. For all accessible temperatures, the *F*_*N*_ pathway significantly dominates. The *F*_*C*_ folding pathway is more populated at lower temperatures and in the presence of confinement. At the highest accessible folding temperature in bulk (112), probabilities of the observed *F*_*C*_ pathway are equal to 2% (bulk) and 18.5% (confinement).

**Table 2:** Probabilities of (un)folding pathways in different conditions. $P_{F_{N}}$ and $P_{F_{C}}$ denote the folding probability of 3IRT via pathways *F*_*N*_ and *F*_*C*_ at different temperatures *T*. *P*_misfold_ denotes the probability of misfolded structures during the folding of 3IRT. $P_{U_{N}}$ and $P_{U_{C}}$ denote the unfolding probabilities of 3IRT via pathways *U*_*N*_ and *U*_*C*_.

Folding							Unfolding				
*T*	$P_{F_{N}}$ (%)		$P_{F_{C}}$ (%)		*P*_misfold_ (%)		*T*	$P_{U_{N}}$ (%)		$P_{U_{C}}$ (%)	
	bulk	conf	bulk	conf	bulk	conf		bulk	conf	bulk	conf
105	82.0	50.5	14.0	18.0	4.0	31.5	118	96.5	–	3.5	–
107.5	89.5	–	9.5	–	1.0	–	120	–	92.0	–	8.0
110	97.5	75.5	2.5	16.5	0.5	8.0	122	–	94.0	–	6.0
112	98.0	80.0	2.0	18.5	0.0	1.5	125	97.5	97.5	2.5	2.5
115	–	85.5	–	14.0	–	0.5	130	87.0	92.0	13.0	8.0
118	–	93.5	–	6.5	–	0.0	135	89.0	88.5	11.0	11.5
120	–	94.0	–	6.0	–	0.0	140	96.0	95.0	4.0	5.0
							145	100.0	99.0	0.0	1.0

The preference for *F*_*N*_ pathway stems from the fact, that the C-terminus forms the *β*-strand, and therefore it is more strongly “pulled” towards its native position, compared to loose N-terminus. This effect can be also quantified in terms of the energy of the states. In our model, the mean potential energy (at a given temperature) of the structure with C-terminus formed (but not N-terminus) is always lower than the mean energy of the state with only N-terminus formed (for details see S1 Appendix, part 2). Therefore, the state with structured C-terminus is thermodynamically more favorable during folding and hence more commonly formed, leading to the higher prevalence of the *F*_*N*_ pathway. The increased probability of *F*_*C*_ pathway at lower temperatures may however indicate, that this pathway is kinetically more accessible. Indeed, for lower temperatures the core of the structure collapses relatively fast, making it hard for C-terminus to thread the loop due to entropic reasons. On the other hand, the N-terminus has to thread a relatively large loop formed by about 60 residues (helices H4-H7). Therefore, if only the helices are not yet attached to the core of the protein (which is commonly the case), threading is more favorable for N-terminus. The presence of the confinement enhances folding by the *F*_*C*_ pathway narrowing the configurational space scanned by N-tail and forcing the N-terminus stay spatially close the loop it has to thread. On the other hand our preliminary results suggest, that too tight confinement forces the helices H4-H7 to form a tertiary structure (attached to the core), reducing the advantage for N-terminus and hindering (but still not forbidding) folding. Hence one can speculate that very tight confinement will again favor the *F*_*N*_ pathway. This could be possibly checked by folding the UCHs in chaperones with different cage size.

#### Knotting probability of UCH-L1 during folding

**Fig 3:**
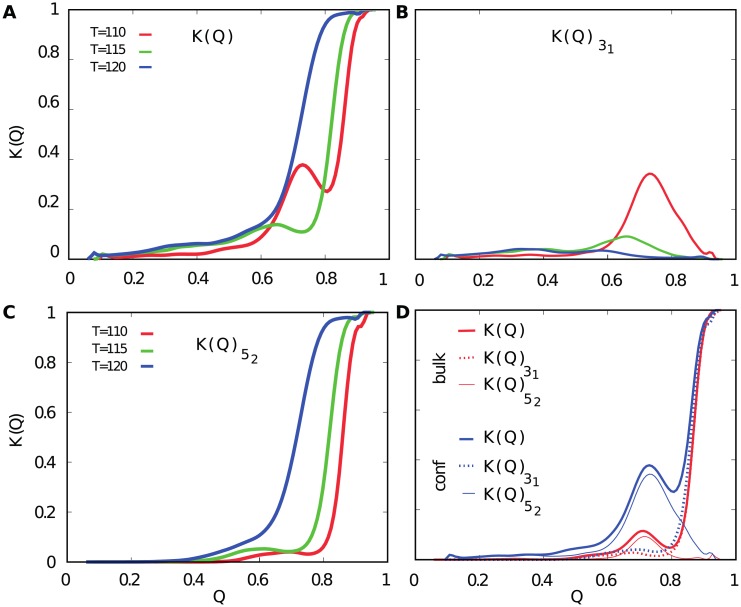
Plots of knotting probability as a function of Q during folding of UCH-L1 protein (PDB code 3IRT) in different conditions and temperatures. A: The total knotting probability *K*(*Q*)_*T*_ (all possible type of knots) in the confinement. B: The knotting probability of a 3_1_ knot ($K{\left(Q\right)}_{3_{1}}$) in the confinement. C: The knotting probability of 5_2_ knot ($K{\left(Q\right)}_{5_{2}}$) in the confinement. D: The comparison of *K*(*Q*), $K{\left(Q\right)}_{5_{2}}$ and $K{\left(Q\right)}_{3_{1}}$ in bulk and the confinement at *T* = 110.

To qualitatively describe topological properties along the folding pathway, we introduce the total knotting probability *K*(*Q*) (as a function of *Q* describing progress in folding). This probability is composed mostly of the probability of 3_1_ ($K{\left(Q\right)}_{3_{1}}$) and 5_2_ ($K{\left(Q\right)}_{5_{2}}$) knots presence. In Fig 3A we show *K*(*Q*) calculated under confining conditions at three different temperatures—below (110) and around (115) estimated *T*_*f*_ for the bulk, and at estimated *T*_*f*_ for the confinement (120). The *T*_*f*_ values are estimated based on the chevron plot described in the following section. The shape of *K*(*Q*) strongly depends on the temperature. Below *T*_*f*_ in the confinement (*T* eq. 110 and 115), there is a local maximum of *K*(*Q*) at *Q* ∼ 0.7, which decreases and shifts to the lower values of *Q* with increasing temperature. Decomposition of *K*(*Q*) on the knot type directly shows that this maximum comes from the probability of the 3_1_ knot ($K{\left(Q\right)}_{3_{1}}$), see Fig 3B. The height of the peak decreases at higher temperatures, which is in accordance with the fact that the *F*_*C*_ pathway (during which temporary 3_1_ is formed) becomes less probable. Finally, at the higher temperature, corresponding to the *T*_*f*_ for confinement, the curve *K*(*Q*) is almost monotonic and almost coincident with the shape of $K{\left(Q\right)}_{5_{2}}$ presented in the Fig 3C. This shows that in the majority of cases, the protein folds through intermediate configuration corresponding to the 5_2_ knot. The difference between *K*(*Q*) and $K{\left(Q\right)}_{5_{2}}$ for low values of *Q* stems from random, short-lived knots discussed in detail in the last section.

The position at which the total knotting probability *K*(*Q*) = 0.5, implies that in the majority of the successful folding routes, knotting is one of the latest steps (*Q* > 0.7 in all cases). With an increasing temperature *K*(*Q*) and $K{\left(Q\right)}_{5_{2}}$ curves shift towards lower values of *Q* (Fig 3A and 3C, S1 Appendix, part 3) as a consequence of overall destabilization of the protein’s structure.

Investigation of all considered knotting probabilities at the temperature close to the *T*_*f*_ for bulk (110) shows that the probability of forming the 5_2_ knot is similar for both conditions (see Fig 3D). Moreover, much more populated *F*_*C*_ pathway in the confinement provides a much larger contribution of 3_1_ knot probability. The introduction of confinement leads to the drastic change of the way how the folding process is realized. On the other hand, the very low probability of folding via the 3_1_ knot in the bulk is postulated by the experimental results [33, 52].

#### Misfolding

**Fig 4:**
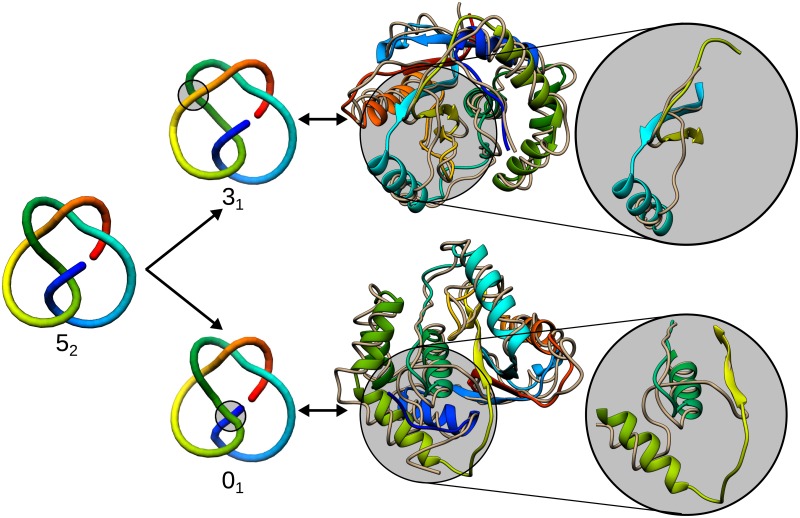
Misfolded structures obtained in UCH-L1 folding. Left: Changing the crossings in 5_2_ knot results either in 3_1_ or 0_1_ knot (the crossings changed are marked with a circle). Middle: Comparison of the native structure of UCH-L1 (rainbow) with its misfolded structure (sandy)—upper panel—3_1_, bottom—0_1_ knot. Encircled is the crucial change in topology. Right: Enlargement of the topology-changing fragment. Note the different arrangement of sandy part of the chain.

The increased probability of *F*_*C*_ pathway at lower temperatures poses a question whether there is a temperature in which the *F*_*C*_ pathway dominates? Such considerations would shed light on the energetic barriers on both folding pathways. However, for low temperature, there is no sufficient thermal energy to break the contacts which formed to fast. This effect is especially evident in the confinement where the confinement enforces the long-distance contacts independently on the proper folding order (Table 2). Nevertheless, in both conditions for temperature close to *T*_*f*_ the fraction of misfolded structures is negligible. The misfolded structures can be divided into two topologically distinct types: 3_1_ knotted (more common) and unknotted (less common) conformations. There is, however, no dominating structure of misfolded product. Some examples are shown in Fig 4. To check whether the misfolded structures are not just artifacts of a too simplified model, we rebuilt the side chains from *Cα* trace using Modeller. We obtained all-atom models with DOPE potential lower than −17.000 suggesting, that the misfolded conformations could be actually found in real conditions (see also S1 Appendix, part 4).

#### Unfolding of UCHs

In our simulations, the unfolding process is, in general, the reversal of the folding, which is again consistent with experimental results [30]. In both conditions (bulk and confinement), unfolding has a common scenario in which an initial destabilization of the native structure results in a significant loss of the tertiary and secondary structure and untying. In particular, we observe two unfolding pathways differing in the occurrence of 3_1_ knotted intermediate. Hence, the unfolding pathways are denoted throughout the text in analogy to their folding counterparts as *U*_*N*_ (direct 5_2_ → 0_1_) and *U*_*C*_ (5_2_ → 3_1_ → 0_1_).

The unfolding process of UCH-L1 is realized mostly by the *U*_*N*_ pathway. Independently of the confining conditions and temperature, we identify more than 85% of unfolding trajectories in which the UCH-L1 unfolded in this way. Similarly to folding, this prevalence comes from higher stabilization of the C-terminus. This explains the fact, that the probability for *U*_*N*_ pathway rises in both conditions, confinement, and bulk, with an increase of temperature up to *T* = 135 (Table 2). Above this temperature, due to thermal fluctuations and unfolding rate, the possible 3_1_ knot probably lives too short to be detected and both termini can be unplugged in comparably short time.

The dependence of unfolding pathway on the knot tails stabilization is also visible comparing different UCHs. Although for all studied proteins, the *U*_*N*_ is the dominating pathway, for UCH-L5 (PDB id 4I6N) with the longest N-terminus, the probability of *U*_*C*_ pathway reaches 39% in bulk at T = 125, which is about 7 times more frequent than in the case of UCH-L1 at the same temperature. On the other hand, the structure with the shortest C-terminus (PDB id 2LEN) has the lowest probability of *U*_*C*_ pathway (for exact numbers see S1 Appendix, part 5).

### Kinetics in the bulk and in the confinement

**Fig 5:**
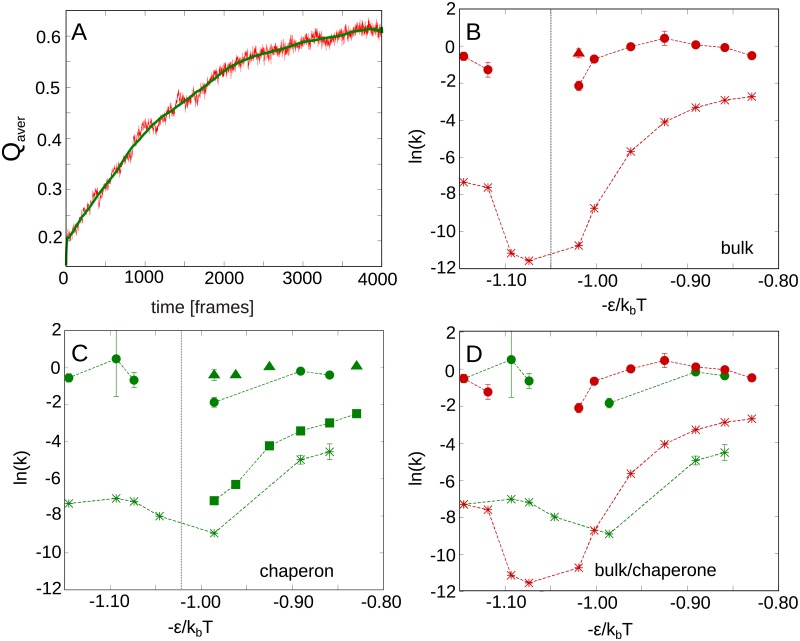
Simulated chevron plot for UCH-L1. A: Representative mean Q as the function of time (red) and smoothed curve (green). B: Chevron plot obtained for bulk. C: Chevron plot obtained for the confinement. B and C comes from fitting od sum of exponentials to plot in A. D: comparison of one slow and one fast phase for bulk (red) and confinement (green). The dashed lines present the expected chevron plot. Fitting the equation describing chevron plot results in an approximate *T*_*f*_ equal 114 (−*ϵ*/*k*_*b*_*T* = −1.05) for bulk and 120 (−*ϵ*/*k*_*b*_*T* = −0.99) for the confinement. In B, C, D the fitting error bars higher than 5% of value were shown.

The UCHs are known to fold and refold along two parallel pathways, each featuring one slow and one fast phase [30, 31, 52]. Therefore, to correlate our model with experimental results we recreated the chevron plot for UCH-L1, with the temperature as a denaturant. In conventional chevron plots, the folding constants are calculated based on the time dependence of e.g. fluorescence. The fluorescence of a protein’s tryptophan depends on its neighborhood. Hence, the fluorescence trace can be understood as a measure of similarity of the tryptophan neighborhood to the native structure. In our simulations, such a measure is given by the fraction of the native contacts—*Q*. Therefore, we calculated the average *Q* as a function of time (representative trace in Fig 5A). The average was taken over all simulations in a given *T* and in each condition (bulk/confinement). Next, we fitted the smoothed *Q*_*aver*_(*t*) with the sum of exponential functions. In particular, we fitted the trace with the sum of the highest number of exponents, for which the fitting errors were lower than 5% of the value. The details of the plot along with the values of obtained constants and errors are presented in S1 Appendix, part 6. Although *Q*_*aver*_(*t*) is only a rough equivalent for the fluorescence, in almost all cases we were able to decompose the trace as a sum of 2-4 exponentials, i.e. to find up to 2 fast and 2 slow phases. These data are shown in Fig 5B (bulk) and 5C (confinement) in the form of chevron plot with the inverse of temperature (precisely −*ϵ*/*k*_*b*_*T*) mimicking the denaturant concentration [53]. For the consistency with conventional chevron plot, we plot the logarithm of *k* = 1/*τ* where *τ* is a characteristic time of a given phase. The obtained values create the trends characteristic for chevron plots, therefore they were connected by dashed lines. Note that in some cases the connection of values is arbitrary. For most cases, the fitting error was an order of magnitude smaller than the value obtained (Fig 5), and those, for which the error was higher (e.g. the point for confinement, −*ϵ*/*k*_*b*_*T* = −1.10), still corresponds to reasonable values.

The presence of the fast and slow phase during folding/unfolding process shows that our results are clearly consistent with previous experimental observations [30, 52]. However, in most cases, we were not able to determine four individual phases (as in the experiment). This may be due to similar characteristic times of separate phases, the model imperfection, or because of a much more complicated folding pathway. Indeed, the curvature of the limbs of the chevron plot indicates more complicated mechanism in each phase, again consistently with the experiment [33]. The detailed analysis of folding/unfolding pathway should be the next step in investigating of these proteins.

To determine the influence of the confinement, we compared the “most complete” kinetic trace for the slow and the fast phases for bulk and confinement (Fig 5D). The slowest phases can be fitted to an equation describing chevron plot, which yields an approximate *T*_*f*_ equal 114 (−*ϵ*/*k*_*b*_*T* = −1.05) for bulk and 120 (−*ϵ*/*k*_*b*_*T* = −0.99) for the confinement (for details see S1 Appendix, part 6). This indicates that confinement stabilizes UCH as it was observed for proteins with trivial topology [44, 54]. Moreover, the chevron plot indicates that the confinement significantly accelerates the folding process, especially the slow phase. In particular, the simulations in the *T*_*f*_ for bulk were not accessible computationally due to very slow rates, while they were accessible in *T*_*f*_ for the confinement. This enables us to calculate near-equilibrium *F*(*Q*) dependence for confinement, which in principle could give additional information on UCHs folding. However in this case, due to the complexity of the folding landscape, the standard ways of its representation do not reveal any new information (S1 Appendix, part 7).

Regardless of the conditions, a collapse of the protein (the first phase of folding) occurs relatively fast, which stays in accordance with the experimental results, that the knotting (occurring in our model for *Q* > 0.7) should be the rate-limiting step [36]. Therefore in our case, the fast phase corresponds to arriving at collapsed, non-knotted form (first part of folding) and the slow phase should correspond to knotting and subsequential reaching of the native structure. The impact of the confinement on the slow phase indicates that the confinement facilitates knotting by restricting the conformational space of the termini. On the other hand, the confinement slows down the unfolding process by slowing down the unknotting—note the change of order in the curves in Fig 5D. The lower unfolding rates in the confinement may be also a result of the *retying* during unfolding (discussed in the next section). As it turns out that the once unfolded knot has a higher probability to retie in the confinement which results in higher knot stability and slower unfolding. Slow unfolding is again in agreement with intuition and experimental observation made for proteins with trivial topology [55].

To additionally investigate the influence of the confinement on both unfolded and folded state, we determined the average asphericity [56] parameter for bulk and confinement in both states. Again, the asphericity of folded state was comparable in both conditions, indicating, that the confinement does not influence the near-folded structures significantly. On the other hand, the asphericity of unfolded state was different in the confinement than in bulk, showing the influence of confinement on the unfolded basin (S1 Appendix, part 8).

### Short-lived and random knots

The probability of knot presence in a polymer chain increases rapidly with its length. As a result, it is highly probable that the sufficiently long polymer will spontaneously form a knot. However, the fraction of knotted proteins is far lower than in the case of equally long polymers [57]. Moreover, the spontaneous self-tying of protein chains in the denaturated state was not reported so far even in the natively deeply-knotted structures [22, 58, 59] or in the case of small knotted proteins in confinement [29]. However, in the case of UCHs we observe a significant fraction of (in most cases short-lived) knots, appearing during folding/unfolding pathway, or in the denaturated state (with *Q* < 0.2).

#### UCHs can be knotted in the unfolded basin

**Fig 6:**
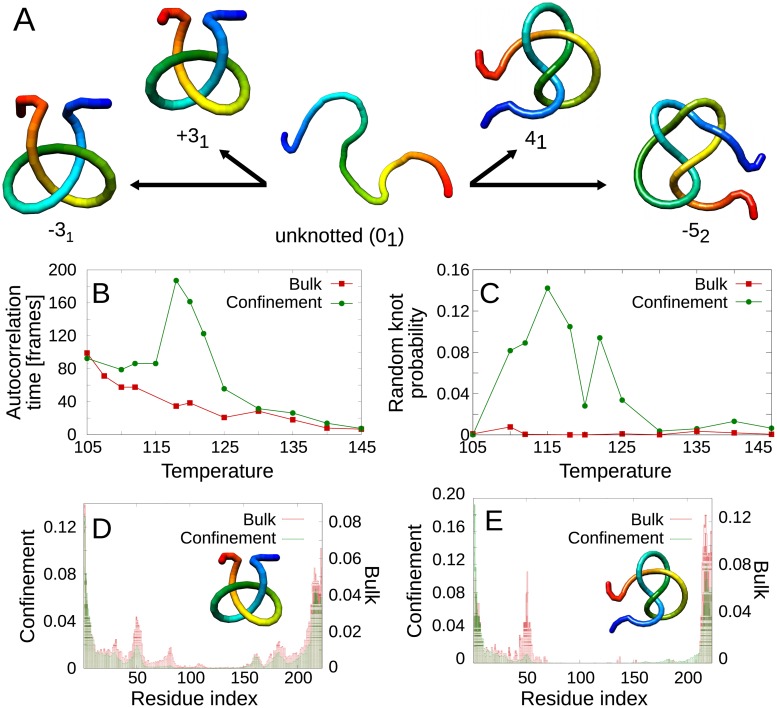
Random knots observed in the unfolded basin of UCH-L1. A: Schematic drawings of the type of observed random knots. B: Autocorrelation time in the unfolded basin as a function of temperature *T*. C: Random knot probability (for details see main text). D: preferred locations of a 3_1_ and E: 4_1_ knot’s termini. In the plots, data for the confinement (green) and bulk (red).

Opposed to other proteins, even deeply knotted ones, in a case of UCHs, in the denatured state we observe various knot types: native-like + 3_1_ and −5_2_ knots, but also non-native −3_1_ and 4_1_ knots (Fig 6A). In most cases, these are short-lived knots (S1 Appendix, part 9) and their probability decreases with knot complexity (in accordance with the theoretical expression for knot probability [60]). To compare the conditions, we calculated the fraction of simulations in which at least one random knot occurred. Similarly to previous results, we observe a much higher number of trajectories with random knots in the confinement. Indeed, in *T* = 120 the random knot in the denaturated state occurred in 92% of trajectories (for raw data on random knots see S1 Appendix, part 9).

The investigation of the number of short-lived knots within a single trajectory is also possible. However, this has to be done with a greater care in order to count only those knots which are not time-correlated. Therefore, at first, we calculated the autocorrelation time as a function of temperature (Fig 6B). This allowed us to calculate the mean number of random knots occurring during the trajectory. In particular, in bulk at low temperatures, there is hardly 1 random knot (in the unfolded state) per 2 trajectories, and at higher temperatures, the probability is even smaller. On the other hand, in the confinement, there are up to 4 random knots on average in one trajectory for *T* = *T*_*f*_. The temperature dependence of these two effects (number of random knots per trajectory and number of trajectories with random knots) are however strictly connected to the time the protein spends in the unfolded basin. To quantify that effect, we calculated the total time the protein is unfolded at a given temperature and divided it into pieces of the length of autocorrelation time for given temperature. Next, we calculated what is the fraction of the number of intervals, in which the protein was knotted, compared to the whole number of such intervals at a given temperature. This quantity we will further call the random knot probability and we present the results in Fig 6C.

Again, the probability of knot formation is significantly larger in the confinement and is highest close to *T* = *T*_*f*_. Under such conditions, the temporary conformations are relatively stable (in contrary to high temperatures), and the protein can search larger part of conformational space before folding (in contrary to lower temperatures). Furthermore, the confinement favors more complex knots. There is only a small number of cases where the 4_1_ or 5_2_ knot occurs in bulk, while there is a meaningful probability of a 4_1_ and a considerable number of 5_2_ knotted structures in the confinement (for examples see S1 Appendix, part 9). This stays in accordance with the similar behavior of polymers, for which the confinement favors more complex knots [61, 62].

To check the geometry of the short-lived knots we determined the preferred location of a knot’s ends (Fig 6D and 6E). It turned out that the confinement favors shallow knots (both 3_1_ and 4_1_), while in the bulk there is a noticeably higher fraction of structures with a deeper knot, with the N-tail threaded by approximately 50 residues. Such knots occur by interaction of helix H8 (indices 193-208) with *β* strand B2 (46-54), with the formation of the twisted loop. This loop may be threaded by the N-terminus, induced by the interaction with helices H4-H7 (91-147). Depending on the threading direction, the resulting structure may have a 3_1_, or 4_1_ knot topology (examples in S1 Appendix, part 9). The fact that the deep knots occur more often in bulk may be somewhat intriguing as the confinement should favor formation of the loop-forming, long-distance B2-H8 interaction. As that surely is the case, the confinement probably also squeezes the newly formed loop, hindering threading due to entropic reasons.

The closer analysis of the autocorrelation time (Fig 6B) reveals that the confinement induces longer correlation times, however, this effect decreases with rising temperature (Fig 6E). Longer correlation time means that in our simulations, the relative change of the structure is slower in the confinement, than in bulk. On the other hand, folding in the confinement is in general faster, therefore the existence of the confinement either stabilizes the native contacts or hinders approximation of parts of the chain, which do not form such contacts. In order to discriminate between the two, first, we compared the mean probability of contact breaking in the confinement and in bulk (details in S1 Appendix, part 10). It turned out that the probability of native contact breaking is lower in confinement, which means that the contacts are more stabilized in the confinement relative to bulk. To investigate the destabilization of structures, with spatially close pieces of chain, which do not form native contacts, we looked at every pair of sufficiently close beads (less than 6Å) which do not form a contact in the native structure. We checked, how often such pair separates for distance larger than 6Å in-between consecutive frames. It turns out that the probability of such pair separation is similar (for intermediate temperatures), or significantly higher (for larger *T*) in the confinements than in bulk. This proves that the confinement works in both ways—stabilizes the native interaction in the same time destabilizing the structures in which pieces of chains which do not form native contacts are spatially close.

#### Short-lived knots may appear during folding or unfolding

**Fig 7:**
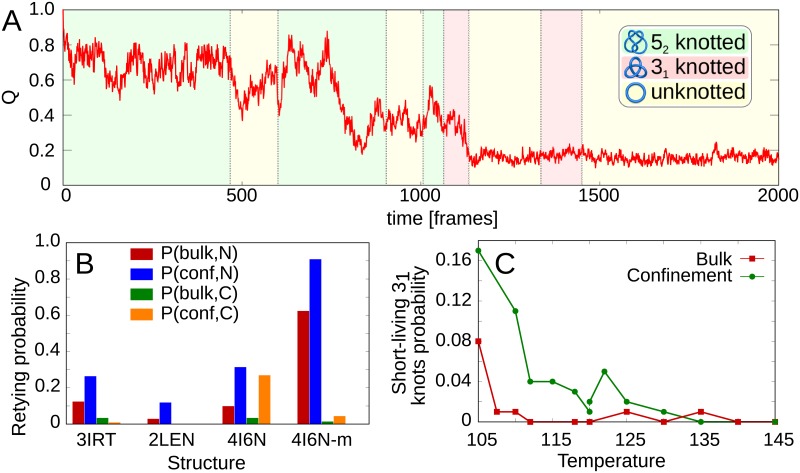
Short-lived knots during folding and unfolding. A: Exemplary unfolding trajectory with the topology indicated by color stripes. B: The retying probability for structures differing in knot tail length. C: The probability of formation of short-lived knots on folding/unfolding pathway. For the confinement at *T* = 120 bottom dot denotes short-lived knots on folding, top—unfolding pathway.

During unfolding, the spontaneous *retying* phenomena can be frequently observed. By *retying*, we define an event, when the partially unfolded, untied protein with the still preserved native-like bias reestablishes the knot as shown in Fig 7A. All the investigated UCHs can retie to a 3_1_ or 5_2_ knot. The *retying* probability, *P*_*retie*_ of a protein, depends on the tails length (Fig 7B, data in S1 Appendix, part 11). For example, the structure with the longest C-terminal knot tail (UCH-L1, PDB code 2LEN) exhibits the probability of retying by C-terminus equal to zero in both conditions. Similarly, the reduction of the N-terminus by 7 residues (from structure 4I6N to 4I6N-m in UCH-L5) significantly increases the N-terminus retying probability. On the other hand, the 3IRT structure (UCH-L1) has the same N-terminus knot tail length as 4I6N-m (UCH-L5), but significantly lower retying probability. This comes from the distribution of native contacts in both structures. The number of the native contacts formed between the N-terminus and its knotting loop for 3IRT (9) is almost two times smaller than for 4I6N-m (17). Those extra native contacts provide an additional force increasing the probability of the retying of 4I6N-m. Independent of the tail length however, the confinement increases the probability of retying. This may also be one of the reasons of deceleration of the unfolding in the confinement (compare Fig 5D), and therefore increased knot stability.

The short-lived knots occur also on a folding pathway. This may be especially bothering, as a non-natively knotted structure may be prone to oligomerization, causing in turn serious neurodegenerative diseases [33]. On the folding pathway, we observed an existence of temporary 3_1_, 4_1_ and 5_2_ knots, which untie after a short time period. The 3_1_ and 5_2_ knots are usually a result of the premature placement of N- or C-terminus in its native position, in which (in early phases of folding) the proper terminus is not stabilized enough, therefore it can slip out of the loop. Consequently, the 3_1_ short-lived knots are more common in confinement and at low temperature, as in such conditions the *F*_*C*_ pathway is more probable.

The 4_1_ knot on the folding pathway occurs rarely. Consistently with the findings presented above, this type of a knot is present more frequently in confinement at low temperatures—3% of folding pathways contain a short-lived 4_1_ knot in the confinement at *T* = 105. For higher temperatures the probability of obtaining a 4_1_ knot diminishes, being negligible for *T* = *T*_*f*_ in both conditions. The 4_1_ knot is very shallow and occurs usually by threading a loose N-terminus through the loop formed by unstructured helices H4-H7 (91-147), similarly as for random knots in the denaturated state.

### Conclusions

We found that confinement leads to faster and more efficient folding of UCH proteins for two reasons. First, encapsulation provides the possibility to fold via an alternative pathway. More precisely, the confinement facilitates folding via the trefoil knot (the *F*_*C*_ pathway) for entropic reasons, while it does not affect folding via the *F*_*N*_ pathway (direct tying, the N-terminus folds the last). This surprising behavior is supported by the experimental observation of uneven influence of chaperonin [63] on the substrate protein rhodanese, which decelerates the folding of the C-terminal domain, but leaves the folding rate of the N-terminal domain unaffected [64]. Second, at the same confinement stabilizes native interactions and destabilizes non-native ones in comparison to bulk, and thus it reduces the height of the free energy barrier and accelerates the folding rate as it was observed for a protein with trivial topology [55, 65, 66]. The two pathways clearly distinguishable in our analysis are in accordance with two pathways identified in experiment [30]. However, there is still no technique, which could determine the topology of the protein during folding *in vitro*, which prevents from direct validation of our results. Some insights can however be given by the study of the tryptophan variants of UCH [33]. In particular, it was shown that the pathways differ in the structure of intermediates for which highly stable central *β*-sheet core and flanking *α*-helices and loop regions are formed differently. This is in accordance with our results, however detailed analysis of folding pathway, with special emphasis put on the location of tryptophan mutations, is required to precisely compare the experimental and theoretical results.

On the other hand, our results show that the confinement introduced by the chaperone-like cage decelerates the unfolding of UCH proteins. Firstly due to a decrease in the effective mobility of the protein backbone upon encapsulation and under topological constrains, which reduce the rate at which new configuration can be explored (especially in twisted loops). This argument without topological contribution is used to explain lower folding rate for a protein with the trivial topology [64]. Secondly, the decelerated unfolding in the confinement is caused by the retying phenomena. It is worth pointing out that the retying phenomena can be used by other knotted proteins with a rather shallow knot, e.g. carbonic anhydrase, to stabilize the structure in a crowded environment (moreover carbonic anhydrase structures with deeper knots also start to be crystallized).

Under the confinement, a significant number of short living knots is observed in the denatured state and in folding and unfolding routes of UCH proteins, what has not been reported for other knotted proteins. More and more complicated knots are more common to occur upon encapsulation, which is in the agreement with the polymer theory [61, 62]. These knots seem to have only positive effect, i.e. their formation accelerates folding. In principle, deeply knotted structures could lead to misfolding, but contrary to the situation in the bulk, they are not formed due to the constrained configurational space.

In summary, we took advantages of structure based model and knot theory, and made the step forward in characterizing folding/unfolding routes for UCH proteins identified experimentally in [30]. We identified possible oligomerization-prone forms of UCHs, which may cause neurodegenerative diseases. We found that weak confinement smooths the rough and not continuous free energy landscape of UCH proteins in a subtle way, e.g. enhancing an indirect tying route. However, at low temperature or strong confinement slower folding should be again observed due to restriction on indirect tying. The deceleration under strong confinement was suggested for a protein with the trivial topology in [63].

## Supporting information

S1 Appendix Supporting tables and figures. (PDF)
