# Supplementary material for: The exclusive effects of chaperonin on the behavior of proteins with 52 knot
Source: PLoS Comput Biol. 2018 Mar 16;14(3):e1005970. doi: 10.1371/journal.pcbi.1005970 (PMC5874080; doi:10.1371/journal.pcbi.1005970)
Supplement: S1 Appendix — (PDF) [file pcbi.1005970.s001.pdf]

Supporting Information

The exclusive effects of chaperonin on the behavior of the 5_2 knotted proteins

Yani Zhao[1,2], Pawel Dabrowski-Tumanski[1,3], Szymon Niewieczerzal[1] and J. I. Sulkowska[1,3]

[1] *Centre of New Technologies, University of Warsaw, Warsaw, Poland*

[2]*Institute of Physics, Polish Academy of Sciences, Warsaw, Poland*

[3]*Faculty of Chemistry, University of Warsaw, Warsaw, Poland*

CONTENTS

I. Structural and sequential comparison of UCHs 3
A. Detailed analysis of investigated proteins: 3IRT, 2LEN, 4I6N, 4I6N-m 3
B. Comparison between different models of 2LEN vs 3IRT, RMSD and number of native contacts 6
C. Modeling the missing loop fragment of the 4I6N 8

II. Mean energy of states 9

III. Influence of temperature on knotting 10

IV. Misfolding and structure rebuilding 11

V. Influence of knot tail lengths on unfolding pathways 13

VI. Kinetics analysis 14
A. Chevron plot 14
B. Determination of folding temperature 14
C. Folding and unfolding times 15

VII. Thermodynamics 16

VIII. Confinement effect on folded and unfolded states 17

IX. Short-living knots 18
A. Life-time and probability of short-living knots 18
B. Exemplary structures of random knots 19

X. Contact breaking probability 23

XI. Retying probability 24

References 24

I. STRUCTURAL AND SEQUENTIAL COMPARISON OF UCHS

A. Detailed analysis of investigated proteins: 3IRT, 2LEN, 4I6N, 4I6N-m

TABLE A. Sequence similarity and RMSD between four proteins considered in the paper: 3IRT, 2LEN, 4I6N and 4I6N-m[a]

PDB id	3IRT		2LEN		4I6N	
	RMSD Å[b]	Identities[c]	RMSD	Identities	RMSD	Identities
3IRT	–	–	1.999(Å)	99%	1.710(Å)	26%
2LEN	1.999(Å)	99%	–	–	2.372(Å)	25%
4I6N	1.710(Å)	26%	2.372(Å)	25%	–	–
4I6N-m	1.710(Å)	25%	2.372(Å)	25%	0.000(Å)	100%

[a] Compared were chains A, and in case of 2len first model.
[b] Calculated using Chimera[4].
[c] Computed by using BLAST[1]

TABLE B. **Sequential and structural similarity between analyzed proteins and other UCHs.**[a]

Name	PDB	ID%[b]	RMSD Å[c]
UCHL1/UCHL3	3irt/1xd3	53	1.042
UCHL1/UCHL3	3irt/2wdt	35	1.297
UCHL1/UCHL5	3irt/3a7s	22	1.602
UCHL1/UCHL5	3irt/4uf5	23	1.870
UCHL1/Yuh1	3irt/1cmx	27	1.661
UCHL1/UCHL3	2len/1xd3	47	2.119
UCHL1/UCHL3	2len/2wdt	33	2.173
UCHL1/UCHL5	2len/3a7s	24	2.294
UCHL1/UCHL5	2len/4uf5	24	2.585
UCHL1/Yuh1	2len/1cmx	25	2.535
UCHL5/UCHL3	4i6n/1xd3	26	1.599
UCHL5/UCHL3	4i6n/2wdt	27	1.421
UCHL5/UCHL5	4i6n/3a7s	54	1.716
UCHL5/UCHL5	4i6n/4uf5	53	1.820
UCHL5/Yuh1	4i6n/1cmx	24	1.738

[a] Compared were chains A, and in case of 2len first model.
[b] Measured with Clustal Omega algorithm[3]
[c] Calculated using Chimera[4].

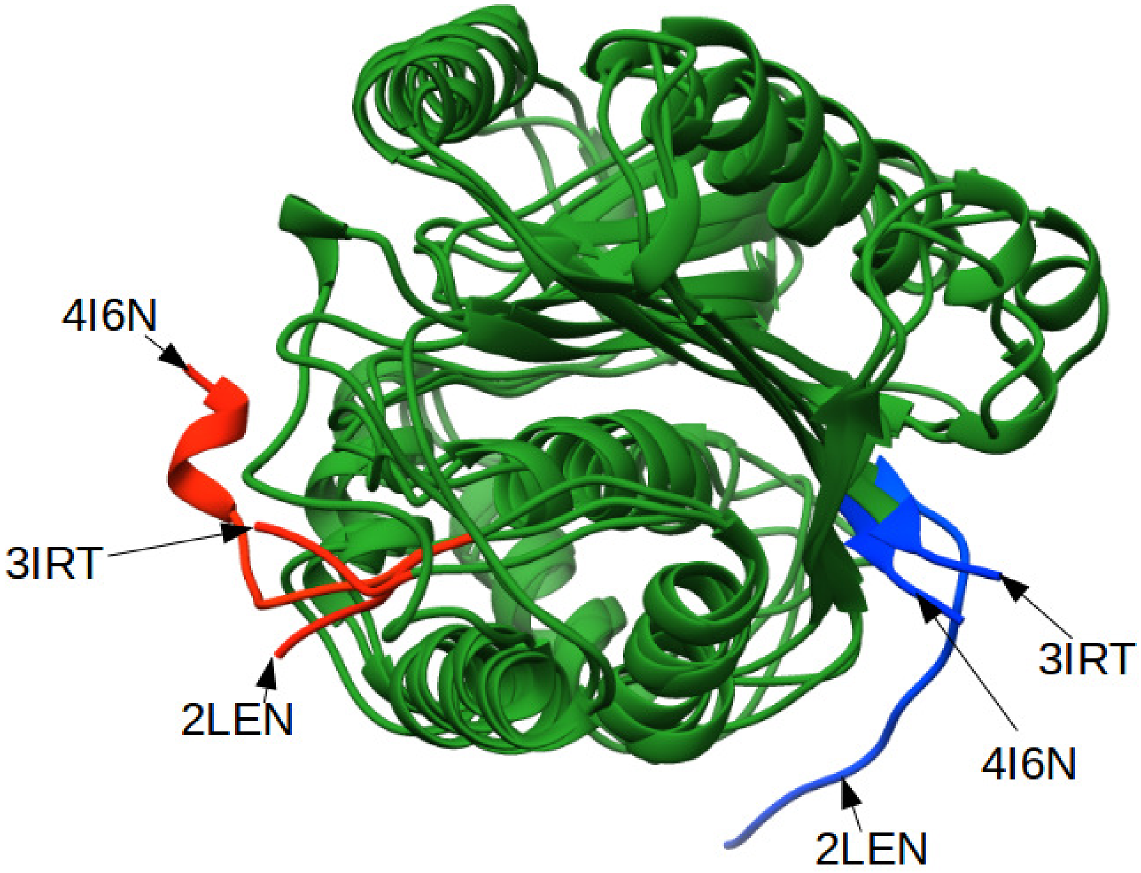

FIG. A. Superposition of the three investigated proteins. The knotting core of proteins is colored green while the N- and C-terminal knot tails are colored red and blue, respectively.

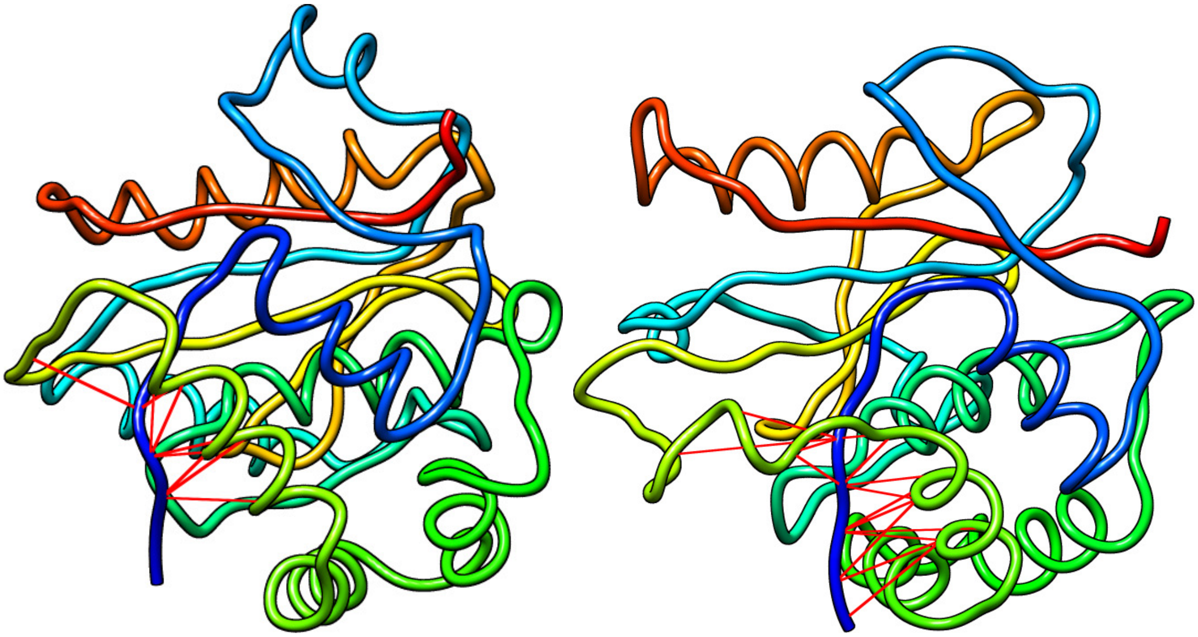

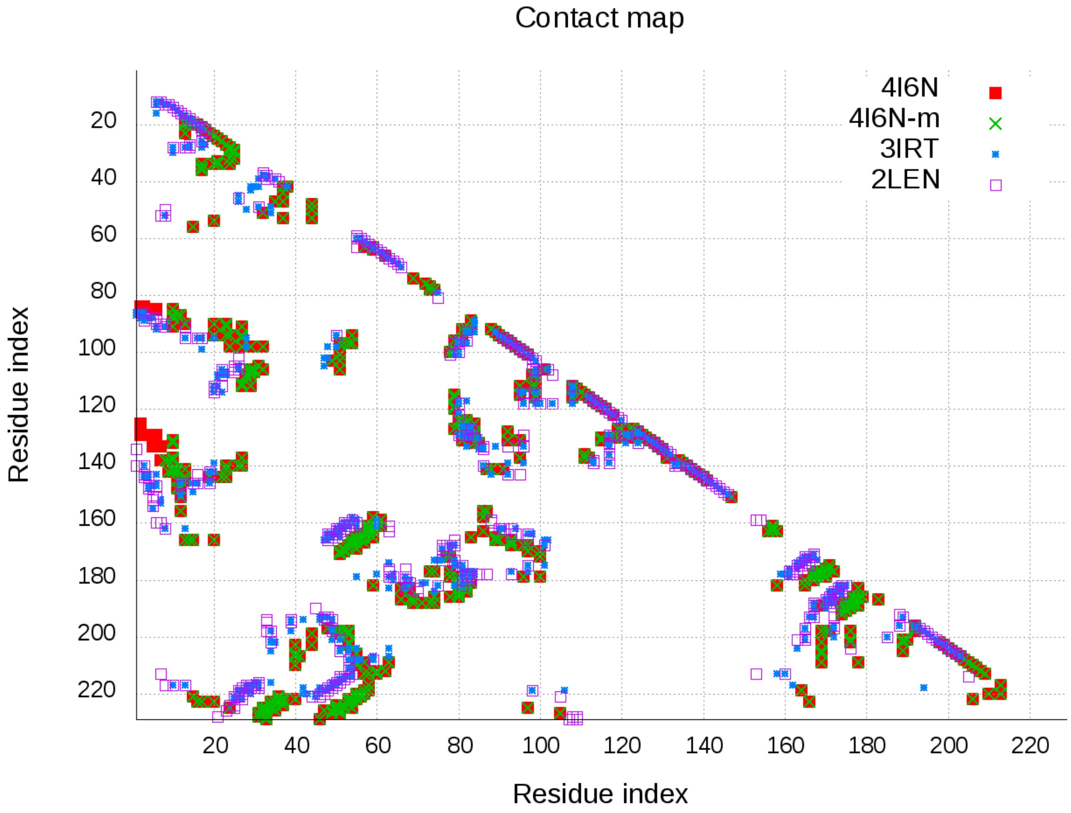

FIG. B. Top: Structures of 3IRT (left) and 4I6N-m (right). Formed native contacts between the N-terminal knot tail (amino acids range: 1-5) and the cross-over loop (amino acids 130-161) are showed with red solid lines for both structures. There exist 9 native contacts for 3IRT and 15 for 4I6N-m. Bottom: the full contact maps of all investigated proteins.

B. Comparison between different models of 2LEN vs 3IRT, RMSD and number of native contacts

TABLE C. RMSD between 3IRT and different 2LEN models (MDL*), and the number of native contacts for 20 2LEN models at different maximum contact distance d. The model used in this work is the first one listed in the PDB file. Although this model has a very close sequence similarity with 3IRT, the difference between the number of their native contacts is 67, even though RMSD is only 2 Å. To check whether this big native contacts difference with 3IRT is a common feature for all 2LEN models, we analysed the number of their native contacts with different cutoffs d_{cutoff} by using the standard parameters proposed by the SMOG server and the RMSD between 3IRT and all 20 models of 2LEN. The obtained results for 2LEN are showed in Table C. For $d = 4.3$ Å, protein 3IRT has 441 native contacts, and it has 701 native contacts when $d = 6.0$ Å. The first model of 2LEN represents well all other models.

PDB id	RMSD (3IRT)	d=6.0 Å	d=4.3 Å
MDL1	1.999(Å)	698	380
MDL2	2.007(Å)	665	376
MDL3	1.995(Å)	670	359
MDL4	2.126(Å)	689	374
MDL5	2.107(Å)	680	366
MDL6	2.207(Å)	694	380
MDL7	2.114(Å)	668	350
MDL8	2.021(Å)	683	365
MDL9	2.201(Å)	668	349
MDL10	2.132(Å)	671	368
MDL11	2.070(Å)	683	389
MDL12	2.082(Å)	657	360
MDL13	1.966(Å)	657	354
MDL14	1.886(Å)	688	379
MDL15	2.052(Å)	673	371
MDL16	2.034(Å)	680	353
MDL17	2.042(Å)	677	375
MDL18	1.993(Å)	680	370
MDL19	1.970(Å)	671	361
MDL20	1.929(Å)	688	362

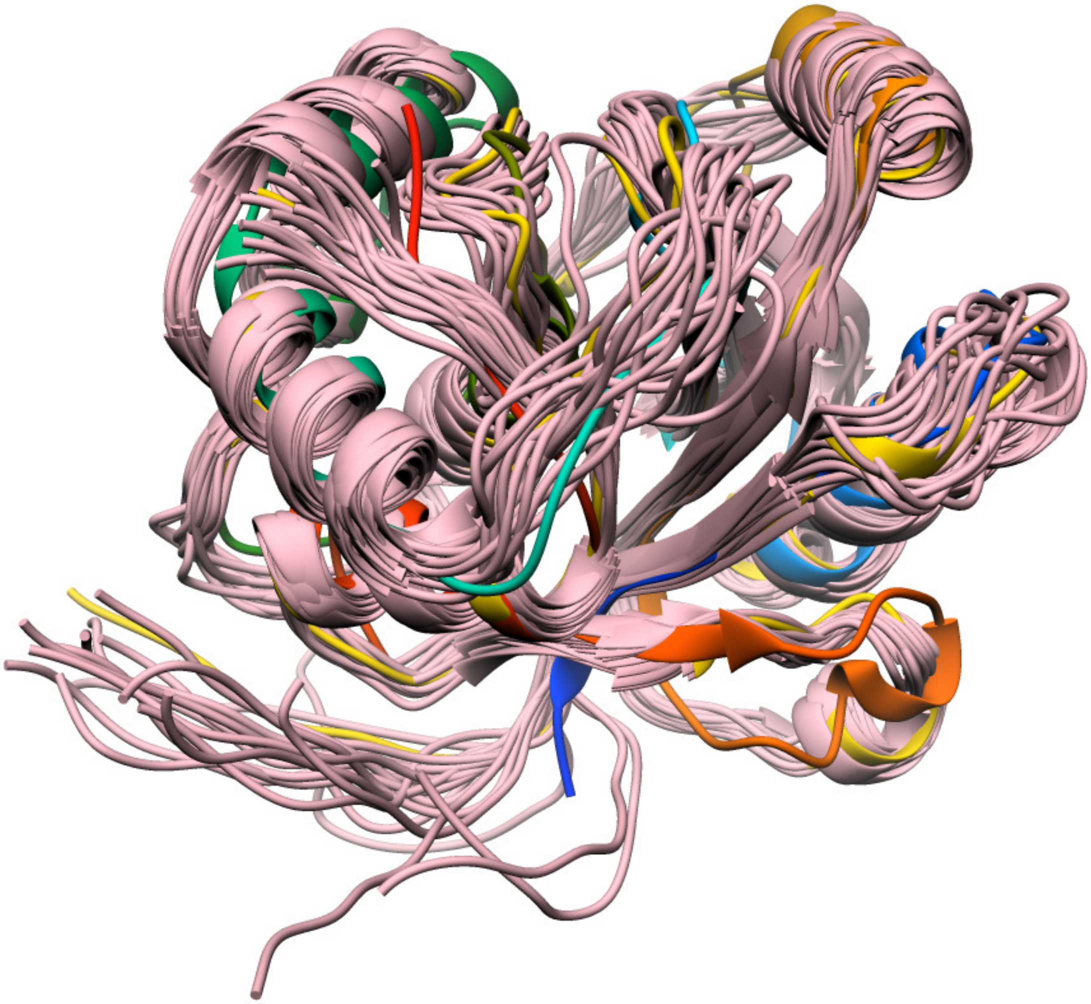

FIG. C. Superimposition of all models of 2LEN (pink color) and 3IRT (rainbow color). The first model studied in this work is denoted with yellow color.

C. Modeling the missing loop fragment of the 4I6N

The secondary structure of 4I6N has a eleven amino acids long gap. The missing sequence of amino acids 142 to 152 belongs to the mobile part of 4I6N, the so called cross over loop[2]. The missing amino acids were added using Modeller. The best conformation, based on the Discrete Optimized Protein Energy (DOPE) calculations, was used in the further studies as a representative of 4I6N, and as a template for 4I6N-m.

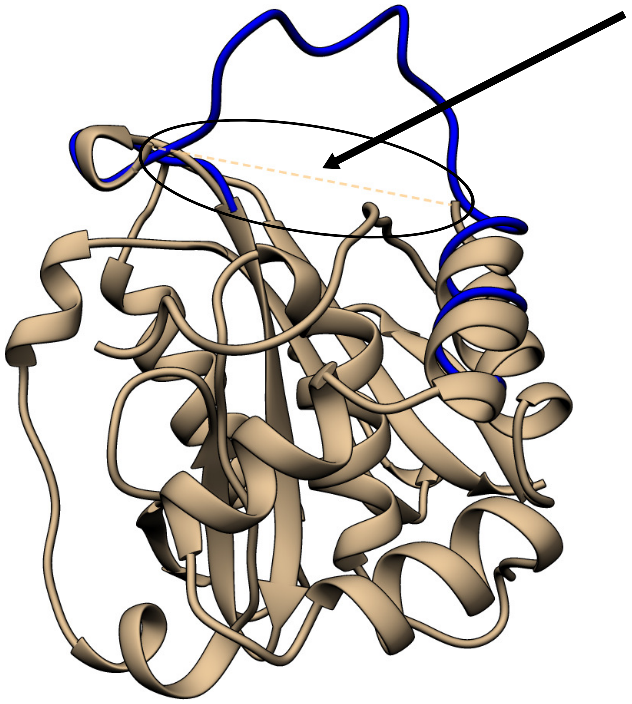

FIG. D. Structure with PDB code 4i6n with its missing fragment inserted (blue).

II. MEAN ENERGY OF STATES

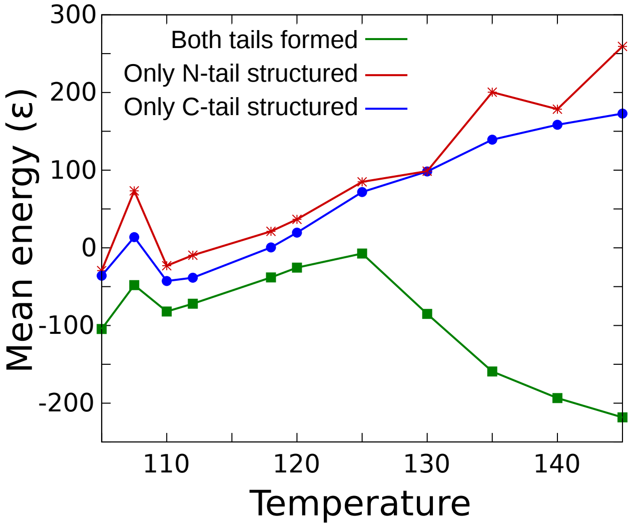

FIG. E. Dependence of mean potential energy of native state (green curve), structure with only C-terminus structured (blue) and only N-terminus structured (red). For high temperatures (unfolding) the protein stays in the native state only in the begging of simulation, therefore only the most native-like structures are included in the mean, resulting in decrease of green curve for higher temperatures.

III. INFLUENCE OF TEMPERATURE ON KNOTTING

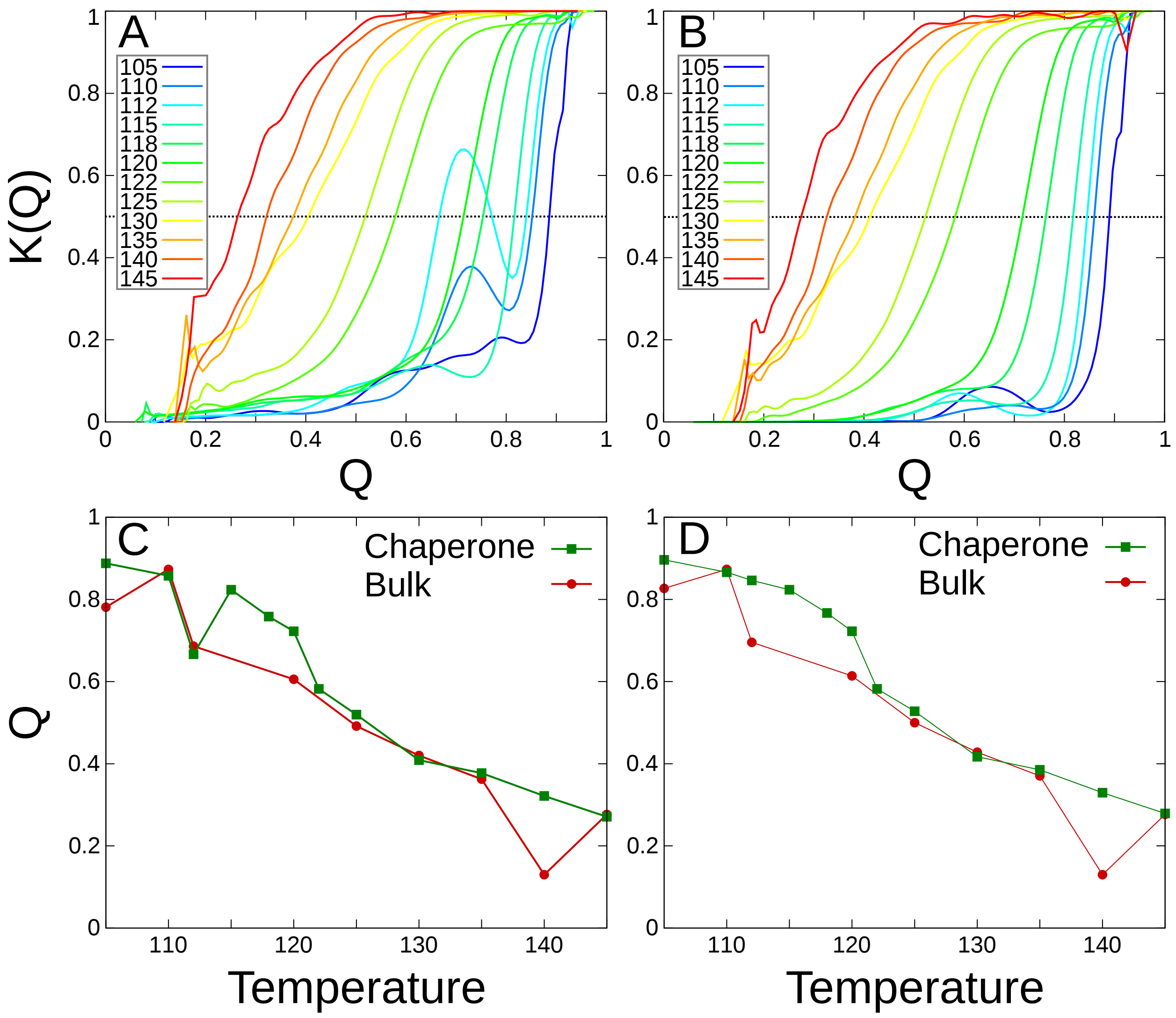

FIG. F. Influence of temperature on knotting. A: The total knotting probability $K(Q)$ for confinement in different temperatures and B: probability of 5_2 knot ($K(Q)_{5_2}$) in different temperatures. Note, that the curves shift towards lower values of Q with rising temperature. This can be quantified by calculating $Q_{0.5}$ for which $K(Q_{0.5}) = 0.5$ (black, dashed line). C: Temperature dependence of $Q_{0.5}$ for which $K(Q_{0.5}) = 0.5$. D: Temperature dependence of $Q_{0.5}$ for which $K(Q_{0.5})_{5_2} = 0.5$.

IV. MISFOLDING AND STRUCTURE REBUILDING

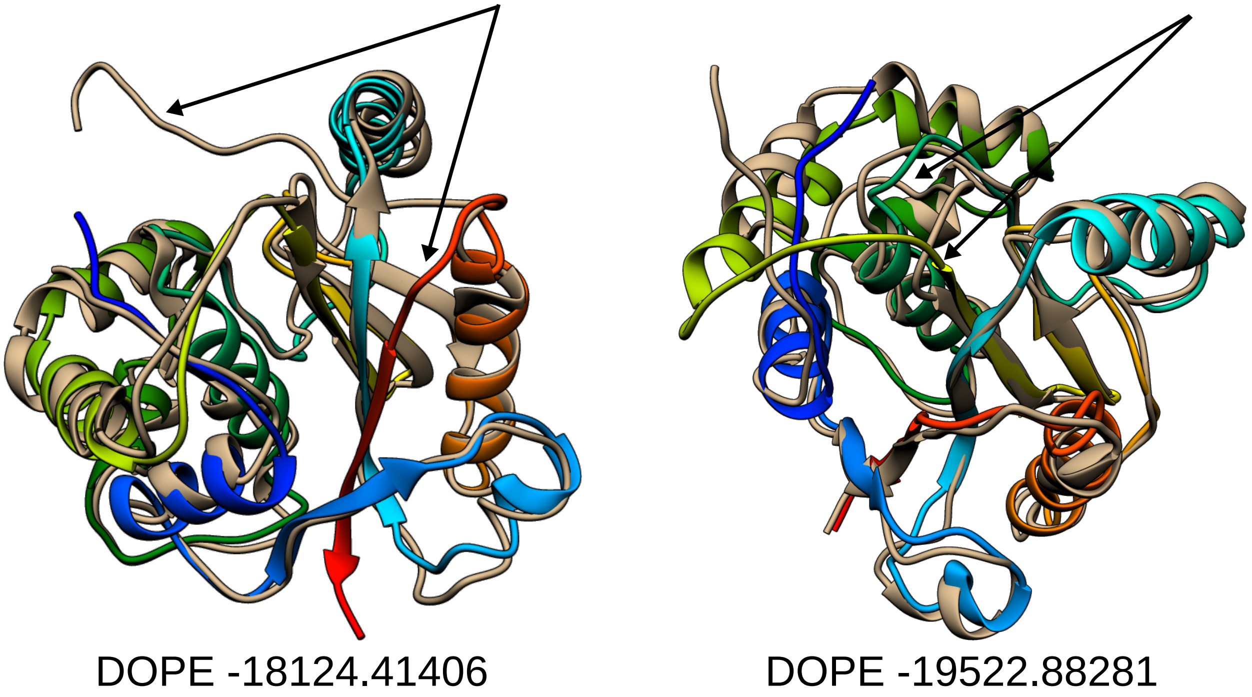

FIG. G. Exemplary misfolded structures with 0_1 (unknot) topology. The misfolded structure from simulations (sandy) was rebuilt from $C\alpha$ trace and overlayed with the crystal structure of 3IRT (rainbow). The arrows show the different arrangement of the chain. Below, the DOPE potential of rebuilt structure is shown.

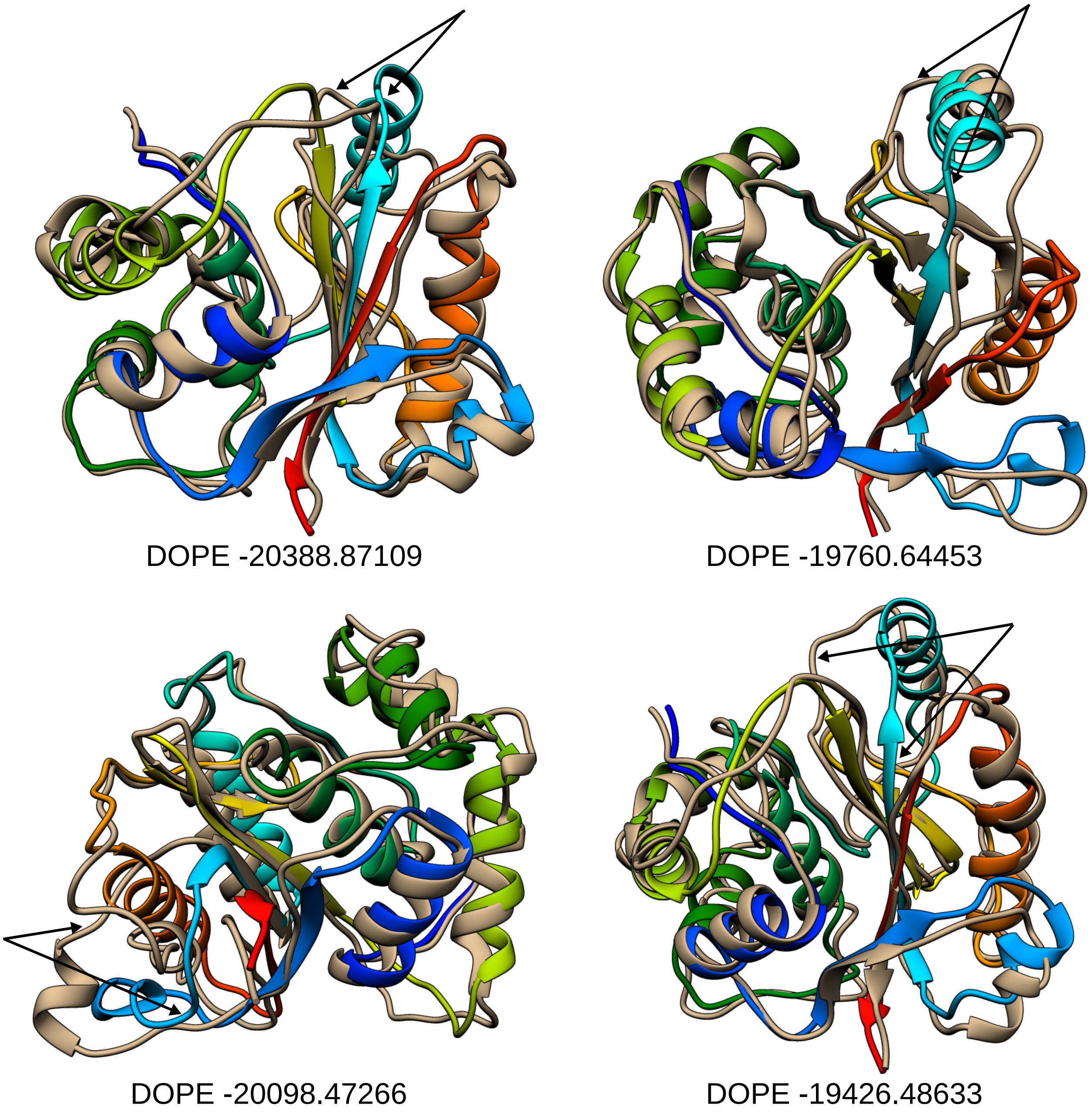

FIG. H. Exemplary misfolded structures with 3_1 (unknot) topology. The misfolded structure from simulations (sandy) was rebuilt from $C\alpha$ trace and overlayed with the crystal structure of 3IRT (rainbow). The arrows show the different arrangement of the chain. Below, the DOPE potential of rebuilt structure is shown.

V. INFLUENCE OF KNOT TAIL LENGTHS ON UNFOLDING PATHWAYS

TABLE D. The probabilities of unfolding pathways for UCHs differing in tail lengths in $T = 125$. For comparison, the tail lengths are given. For description of pathways see main text.

PDB id	N-tail length	C-tail length	U_C[%]		U_N[%]	
			Bulk	Confinement	Bulk	Confinement
3IRT	4	2	5.5	9.0	94.5	91.0
2LEN	5	10	1.0	4.5	99.0	95.5
4I6N	11	3	39.0	27.0	61.0	73.0
4I6N-m	4	3	3.5	6.0	96.5	94.0

VI. KINETICS ANALYSIS

A. Chevron plot

The averaged and smoothed dependencies Q(t) for each given temperature and each condition (bulk/confinement) were used to fit the equation:

$$Q_{smooth}(t) = y_0 + \sum_{i=1}^{n} A_i e^{-k_i t} \tag{1}$$

With all A_i and k_i positive and y_0 being the vertical shift (always close to 0). The number $n \in \{2, 3, 4\}$ of exponential functions fitted was dependent on the fitting errors obtained. We fitted the maximal number of exponential functions, for which the errors were maximally of order of magnitude of obtained parameters. The fitting was done using QtiPlot with unscalled Levenberg-Marquardt algorithm and tolerance 10^{-5}. Obtained values are stored in the Tab. E and F.

TABLE E. Values obtained after fitting of sum of exponential functions in Eq. 1 to the smoothed, averaged $Q(t)$ for bulk conditions.

Bulk									
**Temp.**	$ln(A_1)$	$ln(k_1)$	$ln(A_2)$	$ln(k_2)$	$ln(A_3)$	$ln(k_3)$	$ln(A_4)$	$ln(k_4)$	y_0
105	0.0164576 $\pm 5.933 \cdot 10^{-4}$	7.33657 $\pm 1.910 \cdot 10^{-3}$	-2.08538 ± 2.162	0.556887 ± 0.226	—	—	—	—	-0.0834866 $\pm 6.701 \cdot 10^{-4}$
107.5	-0.179036 $\pm 1.968 \cdot 10^{-4}$	7.62408 $\pm 5.066 \cdot 10^{-4}$	-1.83017 ± 0.321	1.28028 ± 0.396	—	—	—	—	0.0390282 $\pm 1.043 \cdot 10^{-4}$
110	0.7482776 $\pm 6.361 \cdot 10^{-4}$	11.1574 $\pm 8.598 \cdot 10^{-4}$	—	—	—	—	—	—	-1.21954714 $\pm 1.381 \cdot 10^{-2}$
112	0.651057 $\pm 3.091 \cdot 10^{-3}$	11.5691 $\pm 4.636 \cdot 10^{-3}$	—	—	—	—	—	—	-0.920578 $\pm 6.209 \cdot 10^{-3}$
118	-0.0268048 $\pm 7.030 \cdot 10^{-4}$	10.7493 $\pm 1.433 \cdot 10^{-3}$	-2.35784 ± 0.355	2.14529 ± 0.254	-1.0486 ± 0.124	0.397508 ± 0.242	—	—	-0.232521 $\pm 7.751 \cdot 10^{-4}$
120	0.295193 $\pm 3.964 \cdot 10^{-3}$	8.73989 $\pm 6.695 \cdot 10^{-3}$	-1.13872 ± 0.116	0.703687 ± 0.126	—	—	—	—	-0.5353 $\pm 5.735 \cdot 10^{-3}$
125	-0.158162 $\pm 8.810 \cdot 10^{-4}$	5.67912 $\pm 1.249 \cdot 10^{-3}$	-0.832203 $\pm 9.759 \cdot 10^{-2}$	0.037371 ± 0.082	—	—	—	—	-0.0105577 $\pm 5.107 \cdot 10^{-5}$
130	-0.0986317 $\pm 5.002 \cdot 10^{-3}$	4.09644 $\pm 7.166 \cdot 10^{-3}$	-0.61651 ± 0.610	-0.412929 ± 0.383	—	—	—	—	-0.00768915 $\pm 5.655 \cdot 10^{-4}$
135	-0.0979271 $\pm 3.587 \cdot 10^{-3}$	3.31609 $\pm 4.428 \cdot 10^{-3}$	-0.903735 ± 0.133	-0.0638898 ± 0.115	—	—	—	—	-0.00937775 $\pm 2.053 \cdot 10^{-4}$
140	-0.0825264 $\pm 4.590 \cdot 10^{-3}$	2.91755 $\pm 5.046 \cdot 10^{-3}$	-1.07677 ± 0.102	0.0829982 ± 0.105	—	—	—	—	-0.00388177 $\pm 1.724 \cdot 10^{-4}$
145	-0.135801 $\pm 7.222 \cdot 10^{-3}$	2.72562 $\pm 6.584 \cdot 10^{-3}$	-1.05609 $\pm 4.404 \cdot 10^{-2}$	0.519616 $\pm 6.974 \cdot 10^{-2}$	—	—	—	—	-0.00425597 $\pm 1.569 \cdot 10^{-4}$

B. Determination of folding temperature

The most complete trace of Chevron plot were fitted with the equation

$$c(x) = log\left(exp(k_f + m_f x + m_{f^2} x^2) + exp(k_u + m_u x + m_{u^2} x^2)\right) \tag{2}$$

TABLE F. Values obtained after fitting of sum of exponential functions in Eq. 1 to the smoothed, averaged $Q(t)$ for confinement conditions.

Confinement									
**Temp.**	$ln(A_1)$	$ln(k_1)$	$ln(A_2)$	$ln(k_2)$	$ln(A_3)$	$ln(k_3)$	$ln(A_4)$	$ln(k_4)$	y_0
105	-2.86063 ±0.216	7.33657 $\pm1.909 \cdot 10^{-3}$	-0.758791 $\pm5.933 \cdot 10^{-4}$	0.55690 ±0.226	—	—	—	—	-0.653453 $\pm3.086 \cdot 10^{-4}$
110	-0.240050 $\pm2.568 \cdot 10^{-3}$	7.06177 $\pm6.604 \cdot 10^{-3}$	-2.53196 ±3.472	-0.474739 ±2.036	—	—	—	—	0.956111 $\pm2.435 \cdot 10^{-3}$
112	-0.430998 $\pm2.520 \cdot 10^{-3}$	7.22836 $\pm5.677 \cdot 10^{-3}$	-3.14735 ±0.378	0.679557 ±0.411	—	—	—	—	0.828762 $\pm1.912 \cdot 10^{-3}$
115	-0.416120 $\pm4.556 \cdot 10^{-4}$	8.01943 $\pm8.453 \cdot 10^{-4}$	—	—	—	—	—	—	-0.03102 ±0
122	-0.520242 $\pm2.265 \cdot 10^{-3}$	8.94098 $\pm4.496 \cdot 10^{-3}$	-1.80692 $\pm9.994 \cdot 10^{-3}$	7.18765 $\pm1.360 \cdot 10^{-2}$	-2.18795 ±0.429	1.87322 ±0.261	-1.23698 ±0.137	0.409357 ±0.294	1.24517e-06 $\pm5.741 \cdot 10^{-4}$
125	-0.226241 $\pm9.388 \cdot 10^{-4}$	6.30772 $\pm1.363 \cdot 10^{-3}$	-0.882967 $\pm7.588 \cdot 10^{-2}$	0.405588 $\pm7.526 \cdot 10^{-4}$	—	—	—	—	-2.3883e-05 $\pm7.285 \cdot 10^{-5}$
130	-0.158497 $\pm2.046 \cdot 10^{-3}$	4.22176 $\pm2.805 \cdot 10^{-3}$	-0.76605 ±0.109	-0.0357839 $\pm9.009 \cdot 10^{-2}$	—	—	—	—	-0.000121833 $\pm1.512 \cdot 10^{-4}$
135	-4.21756 ±0.302	4.97227 ±0.239	-0.163233 $\pm4.697 \cdot 10^{-3}$	3.41977 $\pm8.268 \cdot 10^{-3}$	-0.942395 $\pm6.124 \cdot 10^{-2}$	0.1909934 $\pm6.452 \cdot 10^{-2}$	—	—	$-9.87439 \cdot 10^{-4}$ $\pm2.510 \cdot 10^{-4}$
140	-0.481687 ±0.600	4.54486 ±0.4256	-1.61189 $\pm5.186 \cdot 10^{-3}$	2.98689 $\pm1.028 \cdot 10^{-2}$	-0.996943 $\pm4.164 \cdot 10^{-2}$	0.401327 $\pm5.777 \cdot 10^{-2}$	—	—	$-3.34371 \cdot 10^{-4}$ $\pm1.734 \cdot 10^{-4}$
145	-0.0184903 $\pm5.010 \cdot 10^{-3}$	2.48658 $\pm4.988 \cdot 10^{-3}$	-1.24301 ±0.132	-0.0702766 ±0.130	—	—	—	—	-3.631e-07 $\pm1.311 \cdot 10^{-4}$

where first part describes folding, second unfolding. As the limbs of Chevron plot in the Fig. 5 main text are both curved, we included the second order effect (dependence of x^2). The argument x represents the “concentration of denaturant”, which in our case is the value $-\epsilon/k_bT$. The fitting resulted in parameters given in Tab. G.

TABLE G. Parameters obtained in describing the Chevron plot with Eq. 2.

**Conditions**	m_f	m_{f^2}	$ln(k_f)$	m_u	m_{u^2}	$ln(ku)$
Bulk	-2,211.88	-926.138	-1,334.79	-1,158.62	-676.047	-502.69
Confinement	-1,415.67	-639.034	-800.036	-872.131	-516.007	-378.872

The minimum of fitted curve was found to determine the (kinetic) equilibrium temperature which was used in main text (approximatelly 114 for bulk and 120 for confinement).

C. Folding and unfolding times

TABLE H. The characteristic times for the folding and unfolding processes of 3IRT in different temperatures. τ_{knot} denotes the mean knotting time, τ_Q is the mean time at when the system firstly hits the most probable value of Q in the folded state, and $\tau_{tot} = \langle Q|\tau_{knot}\rangle$ denotes the total folding time of 3IRT. Times for unknotting and unfolding are denoted as τ_{unknot} and τ_{UQ}), respectively. The system is studied in both bulk and confinement, which is represented by 'bulk' and 'chap' respectively in this Table.

Folding							Unfolding				
T	τ_{knot} $[\times 10^3]$		τ_Q $[\times 10^3]$		τ_{tot} $[\times 10^3]$		T	τ_{unknot}		τ_{UQ}	
	bulk	chap	bulk	chap	bulk	chap		bulk	chap	bulk	chap
105	3.126	0.582	3.125	0.557	3.130	0.596	118	122.5	–	34.0×10^3	–
107.5	9.802	–	10.381	–	10.383	–	120	121.0	153.5	6.2×10^3	126.8×10^3
110	28.644	0.957	28.503	0.866	28.649	0.974	122	–	110.0	–	4.4×10^3
112	125.247	1.211	125.073	1.169	125.338	1.319	125	90.0	90.0	315.5	541.5
115	–	2.634	–	2.592	–	2.747	130	52.0	53.0	85.0	99.0
118	–	7.886	–	7.967	–	8.001	135	27.5	28.0	46.0	54.0
120	–	24.337	–	25.159	–	25.688	140	21.0	21.0	28.0	32.0
							145	20.0	20.0	23.0	25.5

VII. THERMODYNAMICS

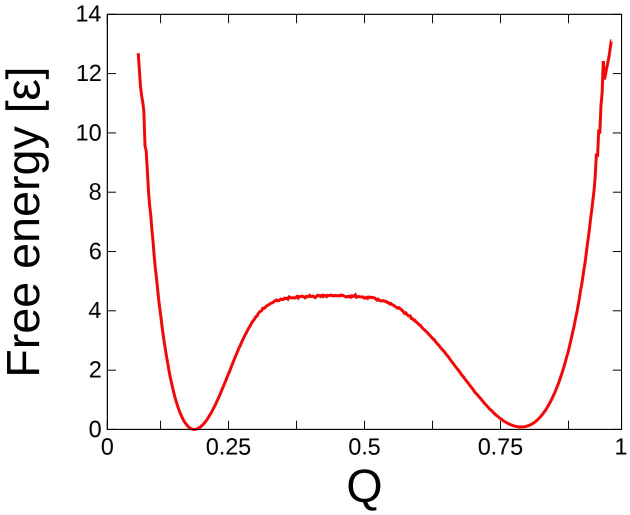

FIG. I. Free energy as a function of Q in the (thermodynamical) equilibrium temperature T=119.6. Note, that this temperature differs slightly from the temperature determined from kinetics. The free energy profile shows broad maximum, which is a convolution of different folding pathways and plasticity of intermediate states.

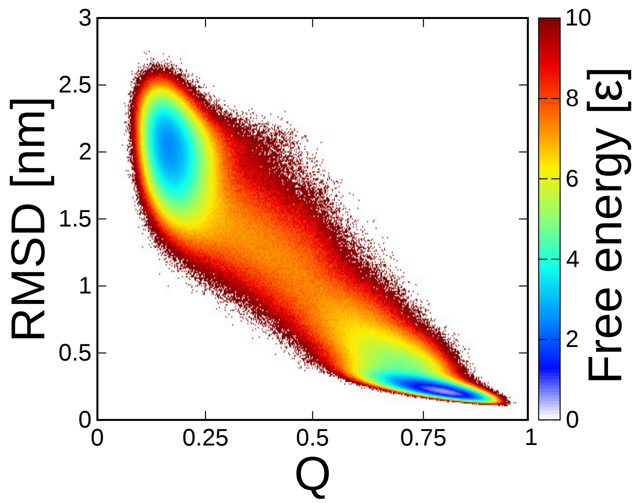

FIG. J. Free energy as a function of Q and RMSD in thermodynamical equilibrium temperature. The landscape suggest one simple pathway connecting the unfolded and folded basins, however the topological analysis reveals at least two pathways, which therefore are indistinguishible in depiction of free energy landscape as the function of standard parameters, such as Q and RMSD.

VIII. CONFINEMENT EFFECT ON FOLDED AND UNFOLDED STATES

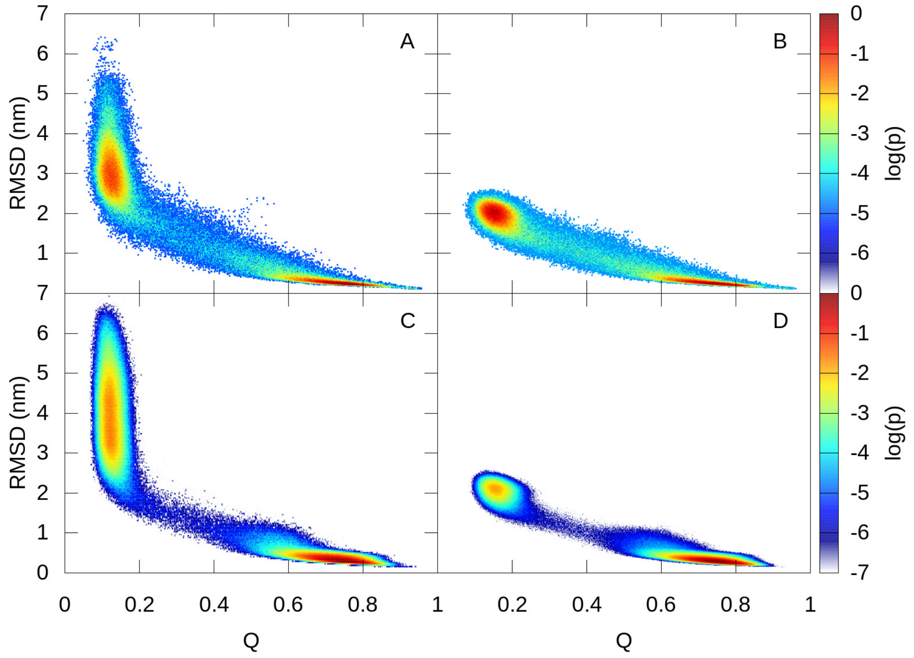

FIG. K. The plot of the logarithm probability distribution log(p) of RMSD (nm) as a function of Q for protein 3IRT (A, B) and 4I6N (C, D) on their unfolding process ($T = 125$) in bulk (A, C) and confinement (B, D).

TABLE I. Asphericity of the unfolded and folded states of 3IRT in bulk and in confinement. Values were calculated at T=110.

bulk		confinement	
Unfolded	Folded	Unfolded	Folded
0.1030	0.0025	0.0141	0.0025

IX. SHORT-LIVING KNOTS

A. Life-time and probability of short-living knots

Most common randomly knotted structures in unfolded state feature 3_1 or 4_1 knot. Probabilities of their occurences (fraction of trajectories in which a random knot occured) are presented in Tab. J and in Fig. L. Tab. J contains also τ_{life} value being the mean life-time of the knot in given condition.

TABLE J. The probabilities of occurences (fraction of trajectories in which a random knot occured) and mean life-time (τ_{life}) of knots in unfolded state.

T	probability						τ_{life}			
	3_1 (%)		4_1 (%)		$\text{tot}_{\text{random}}$ (%)		3_1		4_1	
	bulk	chap	bulk	chap	bulk	chap	bulk	chap	bulk	chap
105	5.5	14.0	0.5	6.0	6.0	18.5	40.4	73.6	17.0	46.9
107.5	9.0	–	1.0	–	10.0	–	74.8	–	10.3	–
110	20.0	18.5	2.5	6.5	21.5	24.0	26.0	80.2	21.0	33.2
112	42.5	28.5	2.0	9.0	43.5	35.5	51.3	27.9	23.0	27.4
115	–	57.5	–	17.5	–	69.5	–	48.3	–	24.1
118	–	77.0	–	29.5	–	79.5	–	50.6	–	32.7
120	–	92.0	–	62	–	92.5	–	61.9	–	29.1

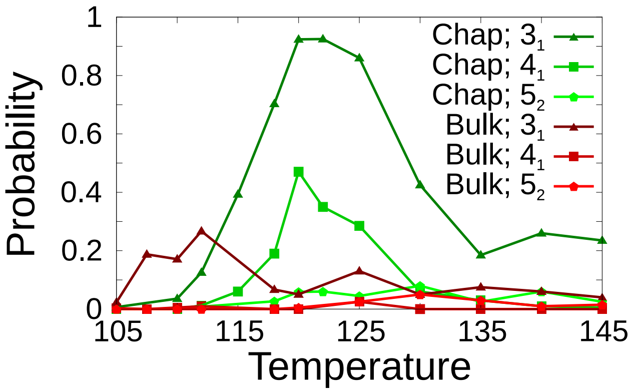

FIG. L. Probability (fraction of trajectories) of occurence a random knot in one trajectory as a function of temperature.

B. Exemplary structures of random knots

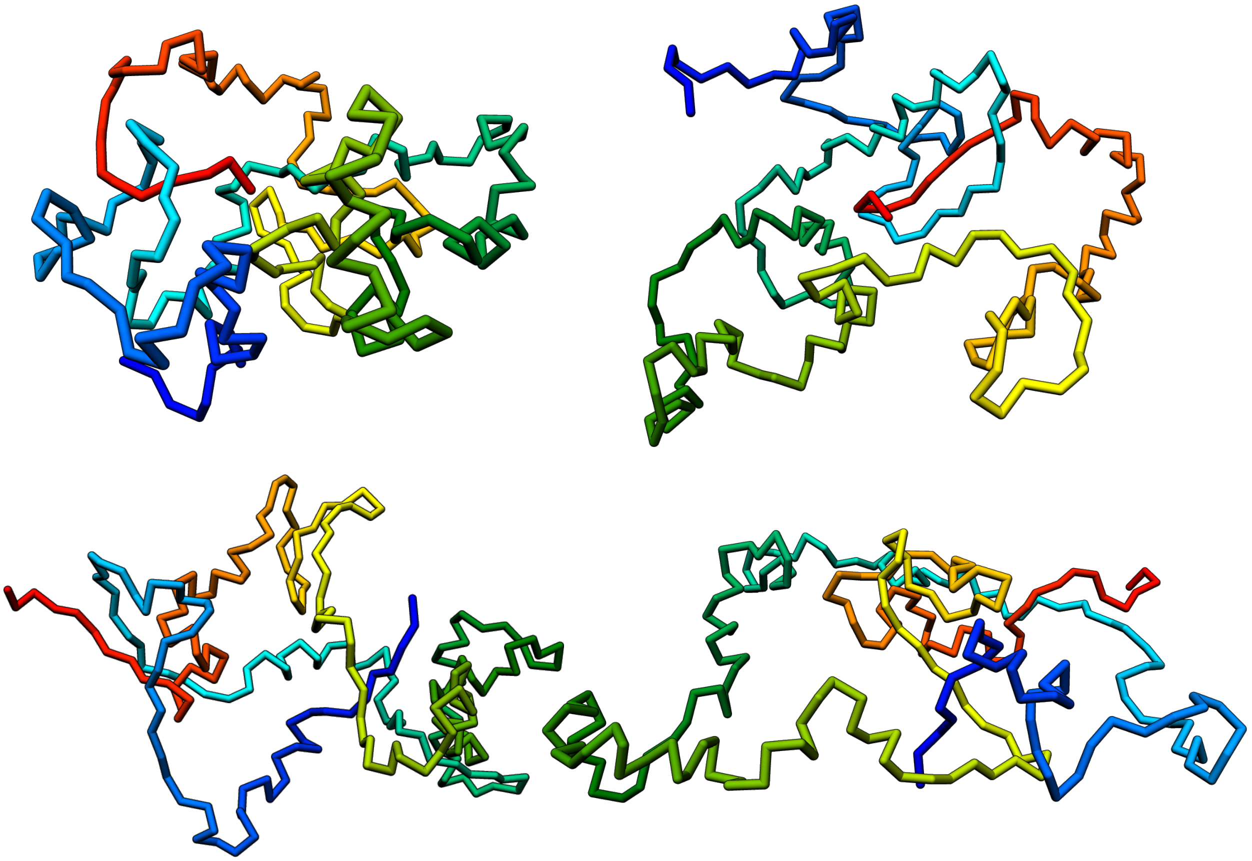

FIG. M. Exemplary structures of unfolded ($Q < 0.2$) UCH-L1 (3IRT) with $+3_1$ topology obtained in simulations.

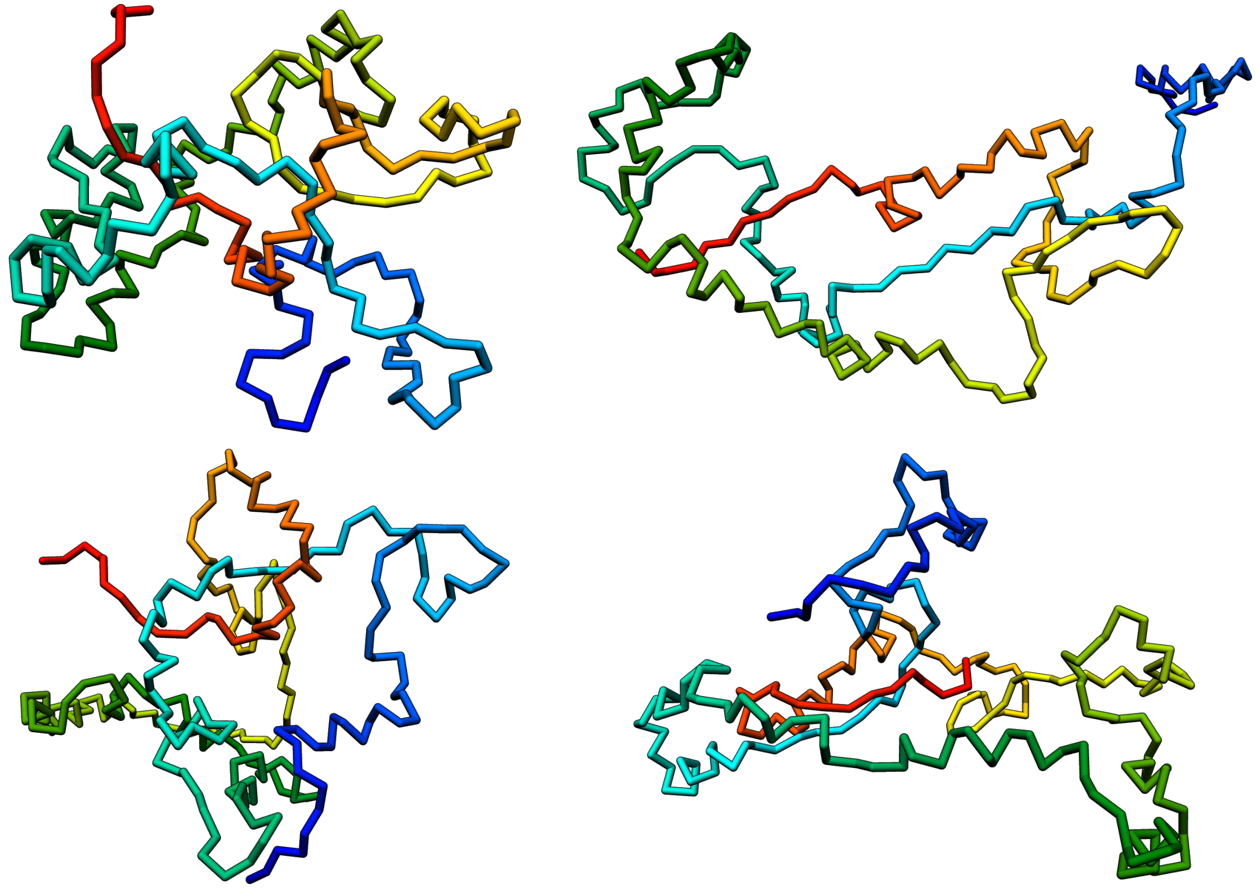

FIG. N. Exemplary structures of unfolded ($Q < 0.2$) UCH-L1 (3IRT) with -3_1 topology obtained in simulations.

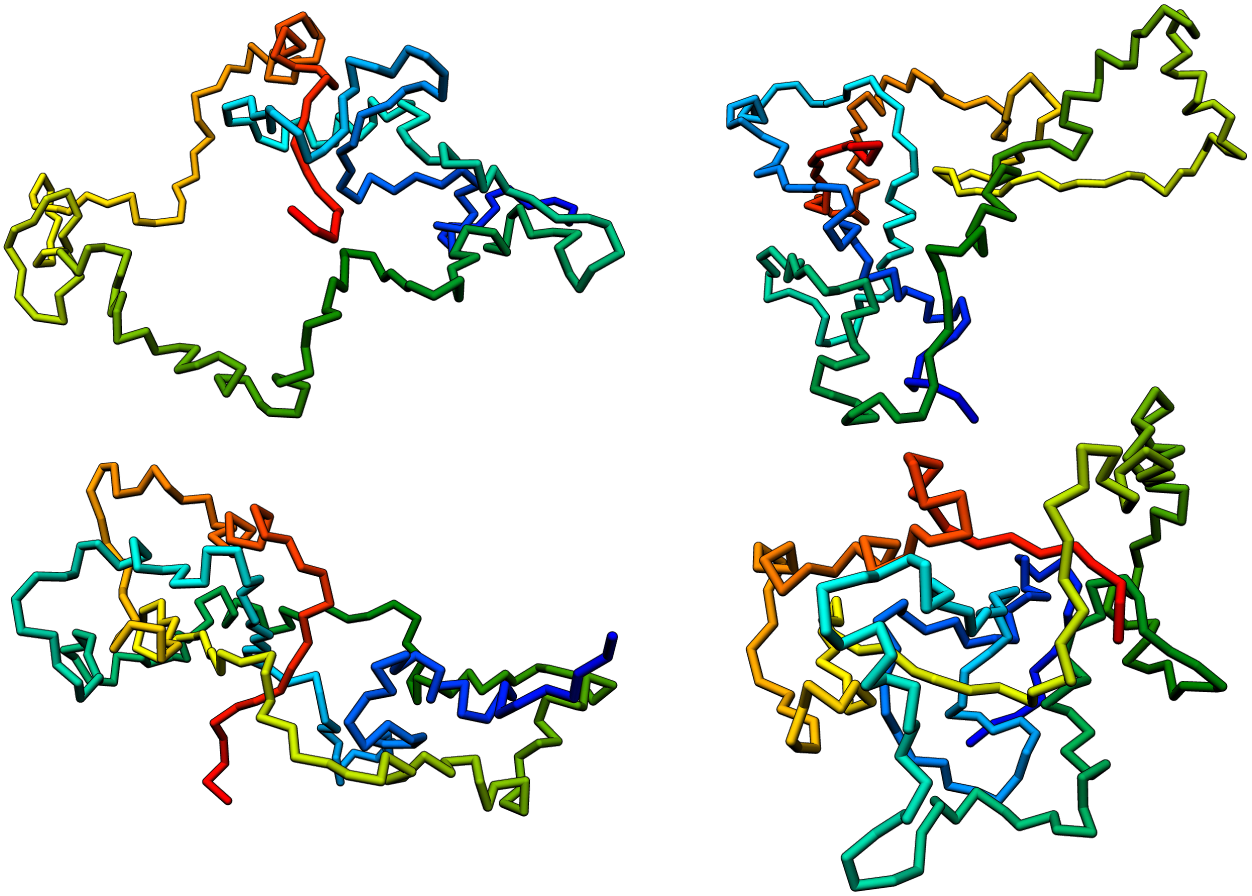

FIG. O. Exemplary structures of unfolded ($Q < 0.2$) UCH-L1 (3IRT) with 4_1 topology obtained in simulations.

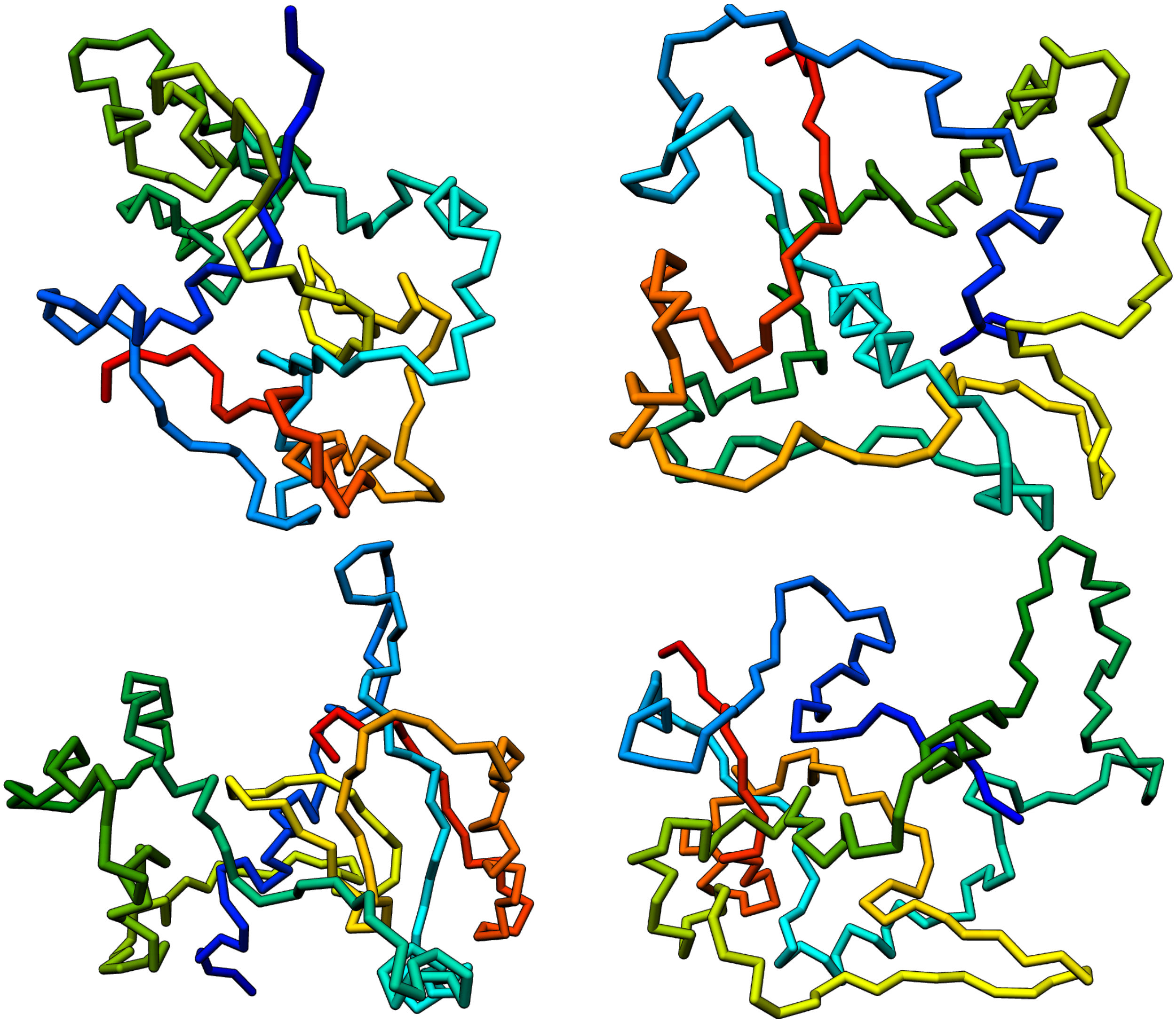

FIG. P. Exemplary structures of unfolded ($Q < 0.2$) UCH-L1 (3IRT) with 5_2 topology obtained in simulations.

X. CONTACT BREAKING PROBABILITY

The contact breaking rate was calculated by comparing the set of contacts between consecutive frames. For each contact (e.g. beetween residues 1 and 5) we identified the frames in which this particular contact was broken, i.e. the situations in which in one frame the contact existed, but it disapeared in the next frames. This number was then divided by the total number of frames in which a given contact existed. This fraction has a meaning of the conditional probability of contact breaking, given that the contact existed. Next, we calculated the mean probability for all contacts as a function of temperature. The same analysis has been done in case of any pairs of beads separated by at least 4 residues, which during simulations were closer than 6Å, but do not form a native contact (in total 23197 such pairs).

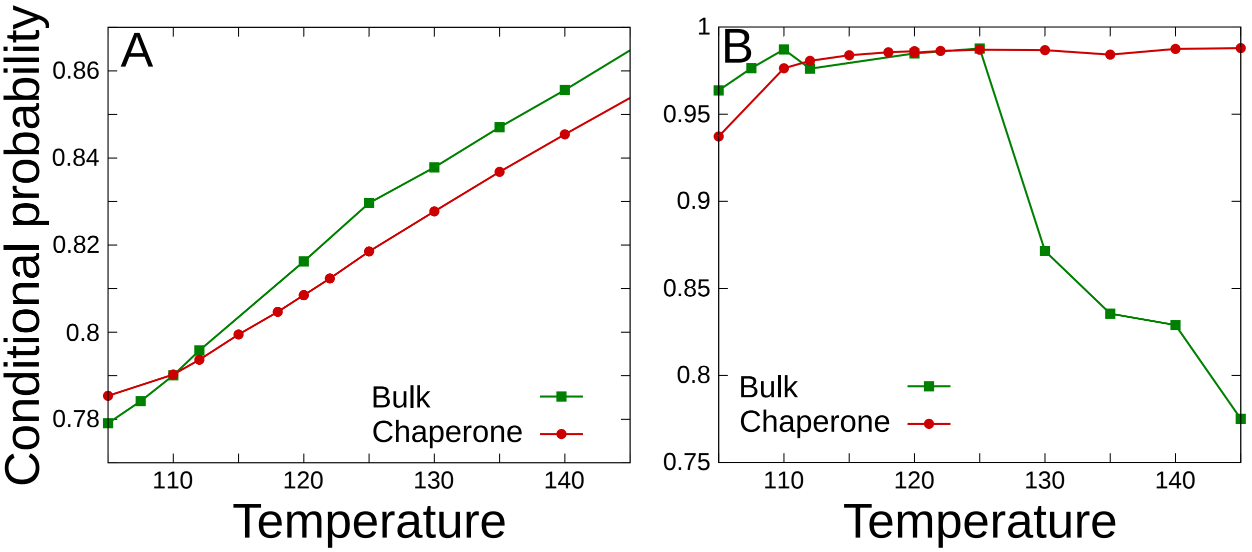

FIG. Q. Mean breaking rate (conditional probability) of contacts as a function of temperature. A: native contacts, B: pairs of beads not forming native contacts. The breaking probability for native contact for temperatures larger than 110 is always higher for bulk, then for confinement, therefore confinement stabilizes native contacts. On the other hand, the separation proability for pairs of beads nt forming native contact is higher for confinement meaning, that confinement destabilizes structures with spatial proximity of beads not forming native interactions.

XI. RETYING PROBABILITY

TABLE K. The retying probability [%] as a function of protein and conditions (bulk/confinement). Data for Fig. 7B in main text. The probability was calculated as a fraction of trajectories in which retying occurs to the total number of trajectories in a given temperature (T=125).

	**3IRT**	**2LEN**	**4I6N**	**4I6N-m**
**Bulk N-tail**	12.5	3.0	10.0	62.5
**Confinement N-tail**	26.5	12.0	31.5	91.0
**Bulk C-tail**	3.5	0.0	3.5	1.5
**Confinement C-tail**	1.0	0.0	27.0	27.0

[1] http://blast.ncbi.nlm.nih.gov/Blast.cgi

[2] Misaghi S, Galardy PJ, Meester WJN, Ovaa H, Ploegh HL, Gaudet R Structure of the Ubiquitin Hydrolase UCH-L3 Complexed with a Suicide Substrate. Journal of Biological Chemistry. 2005;280:1512–1520.

[3] Sievers F, Wilm A, Dineen D, Gibson TJ, Karplus K, Li W, Lopez R, McWilliam H, Remmert M, Söding J et al Fast, scalable generation of high-quality protein multiple sequence alignments using Clustal Omega. Molecular systems biology. 2011;7(1):539

[4] Pettersen EF, Goddard TD, Huang CC, Couch GS, Greenblatt DM, Meng EC, Ferrin TE UCSF Chimeraa visualization system for exploratory research and analysis. Journal of computational chemistry. 2004;25(13):1605–1612
